# Induction of synthetic lethality in IDH1-mutated gliomas through inhibition of Bcl-xL

**DOI:** 10.1038/s41467-017-00984-9

**Published:** 2017-10-20

**Authors:** Georg Karpel-Massler, Chiaki Tsuge Ishida, Elena Bianchetti, Yiru Zhang, Chang Shu, Takashi Tsujiuchi, Matei A. Banu, Franklin Garcia, Kevin A. Roth, Jeffrey N. Bruce, Peter Canoll, Markus D. Siegelin

**Affiliations:** 1 Department of Pathology & Cell Biology, Columbia University Medical Center, New York, NY 10032 USA; 2 Department of Neurosurgery, Columbia University Medical Center, New York, NY 10032 USA; 3 Present Address: Department of Neurosurgery, University of Ulm Medical Center, Ulm, Germany

## Abstract

Certain gliomas often harbor a mutation in the activity center of IDH1 (R132H), which leads to the production of the oncometabolite 2-R-2-hydroxyglutarate (2-HG). In six model systems, including patient-derived stem cell-like glioblastoma cultures, inhibition of Bcl-xL induces significantly more apoptosis in IDH1-mutated cells than in wild-type IDH1 cells. Anaplastic astrocytoma samples with mutated IDH1 display lower levels of Mcl-1 than IDH1 wild-type tumors and specific knockdown of Mcl-1 broadly sensitizes glioblastoma cells to Bcl-xL inhibition-mediated apoptosis. Addition of 2-HG to glioblastoma cultures recapitulates the effects of the IDH mutation on intrinsic apoptosis, shuts down oxidative phosphorylation and reduces ATP levels in glioblastoma cells. 2-HG-mediated energy depletion activates AMPK (Threonine 172), blunting protein synthesis and mTOR signaling, culminating in a decline of Mcl-1. In an orthotopic glioblastoma xenograft model expressing mutated IDH1, Bcl-xL inhibition leads to long-term survival. These results demonstrate that IDH1-mutated gliomas are particularly vulnerable to Bcl-xL inhibition.

## Abstract (web summary)

Glioblastoma (GBM) cells are often characterized by the presence of the IDH1 R132H mutation and high expression of anti-apoptotic proteins. Here, the authors show that the inhibition of Bcl-xL is synthetically lethal in IDH1-mutated GBM models and that this effect is mediated by the oncometabolite, 2-HG, which reduces Mcl-1 protein levels.

## Introduction

Glioblastoma and diffuse gliomas in general remain incurable diseases despite extensive efforts to identify more effective treatment paradigms. The era of personalized medicine has the potential to revolutionize our understanding of malignant neoplasms and to broadly influence therapeutic decision-making. Deep-sequencing technologies have greatly assisted in the identification of novel mutations in cancers. Examples are mutations of IDH1 at codon 132 (R132H) and IDH2 at codon 172 (R172K) in diffuse gliomas and acute myeloid leukemia. The majority of low-grade gliomas and secondary glioblastomas harbor the IDH1 mutation^1^. While glioblastomas are histologically and molecularly heterogeneous, when present, the IDH1 (R132H) mutation is seen in virtually all glioma cells throughout the entire tumor. IDH1- and IDH2-mutated tumors display significantly elevated levels of 2-R-2-hydroxyglutarate (2-HG). While the initial discovery of IDH mutations raised significant excitement in the field, the identification of 2-HG in IDH-mutated tumors received as much attention due to the potential translational implications^2, 3^.

Anti-apoptotic Bcl-2 family members, such as Bcl-xL and Mcl-1, are highly expressed in human glioblastomas and, therefore, it is conceivable that interference with these molecules might exert significant anti-glioblastoma activity. Recent advances in the design of small molecules led to the discovery of BH3-mimetics, such as ABT263. Unfortunately, not all tumors are equally sensitive and it remains pivotal to unravel predictive biomarkers that identify patients with tumors that would especially benefit from the administration/addition of a BH3-mimetic. For example, Mcl-1 is a major mediator of BH3-mimetic resistance.

In this report, we demonstrate that inhibition of Bcl-xL causes synthetic lethality in IDH1-mutated glioblastoma cells in vitro and in vivo and that these effects are mediated by the oncometabolite, 2-HG, which reduces Mcl-1 protein levels. Consistently, our findings reveal that IDH1-mutated gliomas display lower protein levels of Mcl-1.

## Results

### IDH1-mutated glioblastoma cells are more responsive to Bcl-xL inhibition

**Fig. 1:**
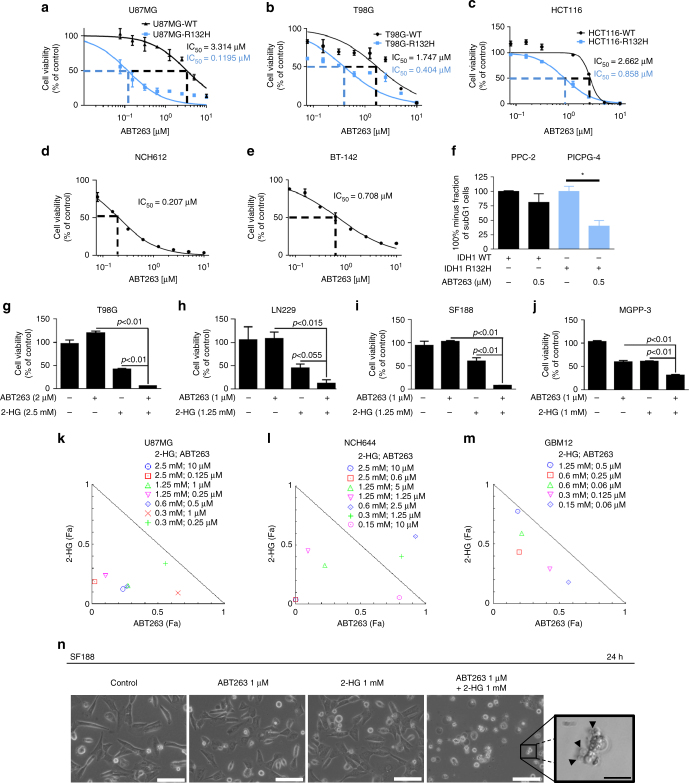
IDH1-R132H-mutated cells are more susceptible to treatment with ABT263. **a** U87MG glioblastoma cells were transduced with pLPCX IDH1-WT or IDH1-R132H prior to treatment with increasing concentrations of ABT263 for 72 h. Cellular viability was determined by MTT assay and the IC_50_-values were calculated based on a non-linear regression analysis. Data are presented as mean and SD, *n* = 3. **b** T98G glioblastoma cells were transduced with pLPCX IDH1-WT or IDH1-R132H prior to treatment with increasing concentrations of ABT263 for 72 h. Cellular viability was determined by MTT assay and IC_50_-values were calculated. Data are presented as mean and SD, *n* = 3. **c** HCT116 IDH1-WT/IDH1-R132H cells were treated with increasing concentrations of ABT263 for 72 h. Cellular viability and IC_50_-values were determined based on MTT assay. Data are presented as mean and SD, *n* = 3. **d**, **e** NCH612 (IDH1-R132H) (**d**) and BT-142 (IDH1-R132H) (**e**) glioma cells were subjected to treatment with increasing concentrations of ABT263 for 72 h. Cellular viability and IC_50_-values were determined by CelltiterGlo assay. Data are presented as mean and SD, *n* = 3. **f** PPC-2 (IDH1-WT) and PICPG-4 (IDH1-R132H) glioma cells were treated for 48 h with solvent or ABT263 as indicated. Staining with propidium iodide was performed and the fraction of viable cells (100%-SubG1 cells) was determined by flow cytometry. Column: mean. *Error bar*, SD (*n* = 3). **g**–**j** T98G (**g**), LN229 (**h**), SF188 **i** and MGPP-3 (**j**) glioblastoma cells were treated for 72 h with solvent, ABT263, 2-HG or the combination of both prior to determining cellular viability by MTT assay. Column: mean. *Error bar*: SD, *n* = 3. **k**–**m** U87MG cells (**k**), NCH644 glioma stem-like cells (**l**) and GBM12 patient-derived xenograft cells (**m**) were treated for 72 h with indicated combinations of ABT263 and 2-HG. Normalized isobolograms were calculated using the CompuSyn software (ComboSyn, Inc., Paramus, NJ). Data points located on the line indicate an additive drug–drug interaction. Data points located below the line indicate a synergistic drug–drug interaction and data points above the line indicate an antagonistic drug–drug interaction. **n** Representative microphotographs are provided for SF188 pediatric glioblastoma cells treated for 24 h with solvent, ABT263 and 2-HG as indicated. *Scale bar*: 100 μm or 20 μm (microphotograph at higher magnification)

Transduced U87MG and T98G glioblastoma cells, bearing the wild-type or mutated form of IDH1 were treated with increasing concentrations of the BH-3 mimetic ABT263, a known inhibitor of both Bcl-xL and Bcl-2. U87MG (IDH1-R132H) cells displayed an approximately thirty times higher sensitivity to ABT263 (IC_50_ = 0.1195 μM—nanomolar range) than their wild-type counterparts (IC_50_ = 3.314 μM) (Fig. 1a). Similarly, in T98G glioblastoma cells treatment with ABT263 resulted in a significantly stronger anti-proliferative response among IDH1-mutated cells moving the respective IC_50_-values into the lower nanomolar range (Fig. 1b).

**Fig. 2:**
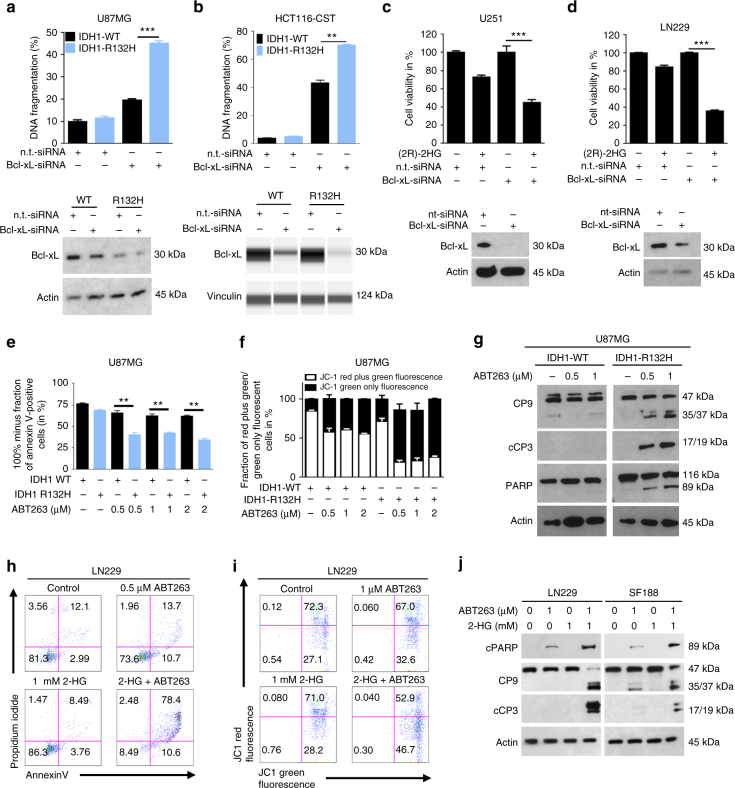
Knockdown of Bcl-xL enhances apoptosis in IDH1-mutated cancer cells. **a** U87MG IDH1-WT/IDH1-R132H glioblastoma cells were transfected either with non-targeting (n.t.)-siRNA or Bcl-xL-siRNA prior to staining with propidium iodide and flowcytometric analysis, *n* = 3. Knockdown of Bcl-xL was confirmed by western blot analysis. **b** HCT116 IDH1-WT/IDH1-R132H cells were transfected either with (n.t.)-siRNA or Bcl-xL-siRNA prior to staining with propidium iodide and flowcytometric analysis. *n* = 3. Knockdown of Bcl-xL was confirmed by capillary electrophoresis. **c** U251 glioblastoma cells were treated with (n.t.)-siRNA or Bcl-xL-siRNA in the presence or absence of 2-HG for 24 h. Staining for propidium iodide was performed. *n* = 3. Bcl-xL knock-down was confirmed by performing western blot analysis for Bcl-xL. **d** LN229 glioblastoma cells were treated with (n.t.)-siRNA or Bcl-xL-siRNA in the presence or absence of 2-HG for 24 h. Staining for propidium iodide was performed. *n* = 3. Bcl-xL knock-down was confirmed by western blot analysis. **e** U87MG IDH1-WT/IDH1-R132H glioblastoma cells were treated for 72 h with increasing concentrations of ABT263 (conc﻿entrations in ﻿μM) or solvent prior to staining with annexin V/propidium iodide and flowcytometry. *n* = 3. **f** U87MG IDH1-WT/IDH1-R132H cells were treated for 72 h with ABT263 or solvent as indicated prior to staining for JC-1 and flowcytometry. A quantitative representation of the fraction of red plus green fluorescent cells vs. green-only fluorescent cells is displayed. *n* = 3. **g** U87MG IDH1-WT/IDH1-R132H glioblastoma cells were treated for 7 h with increasing concentrations of ABT263 or solvent. Whole-cell extracts were collected and western blot analysis was performed for caspase 9 (CP9), cleaved caspase 3 (cCP3), PARP and Actin. **h** Representative flow plots of LN229 glioblastoma cells that were treated for 48 h with solvent, ABT263 and 2-HG as indicated. Staining for annexin V/propidium iodide was performed prior to flowcytometric analysis. **i** Representative flow plots (JC1-staining) of LN229 glioblastoma cells treated for 48 h with solvent, ABT263 and 2-HG as indicated. **j** LN229 and SF188 glioblastoma cells were treated for 7 h with solvent, ABT263 and 2-HG. Western blot analysis is shown for caspase 9 (CP9), cleaved caspase 3 (cCP3), cleaved PARP (cPARP) and Actin. Columns: mean. *Error bars*: SD

To gain further insight on how general this biological observation is we extended our studies to an isogenic model using HCT116 (IDH1-WT/IDH1-R132H) colorectal cancer cells and two patient-derived IDH1-R132H expressing glioma stem-like cells, NCH612 and BT-142. In concordance with our findings in the virally transduced glioblastoma cell lines, HCT116 IDH1-R132H cells (heterozygous knock-in mutation) showed higher sensitivity to ABT263 with a threefold decrease of the IC_50_ value (Fig. 1c). In a similar manner, NCH612 and BT-142 IDH1-R132H-positive glioma stem-like cells showed a high susceptibility toward ABT263 treatment as reflected by nanomolar IC_50_-values (Fig. 1d, e). IC_50_-values for ABT263 in glioma stem-like cells bearing IDH1-WT or IDH1-R132H are displayed in Supplementary Table 1. Next, we addressed the question whether the oncometabolite 2-HG contributed to the ABT263-mediated synthetic lethality seen in IDH1-R132H-expressing cells. U87MG, T98G, LN229, SF188 (pediatric), MGPP-3 (mouse) glioblastoma cells, NCH644 glioma stem-like cells and GBM12 (derived from a patient-derived xenograft) glioma cells were treated with ABT263, 2-HG, the combination of both or vehicle. Across all cell lines tested, the combination treatment resulted in a synergistic anti-proliferative effect (Fig. 1g–n and Supplementary Table 2). To gain a deeper understanding of the molecular mechanism(s) responsible for the preferential response of IDH1-mutated cancer cells to ABT263 treatment, we performed knockdown experiments with small-interfering RNA (siRNA) against Bcl-xL, a known major target of ABT263. Knockdown of Bcl-xL resulted in a significant increase in the subG1 fraction of U87MG IDH1-R132H and HCT116 IDH1-R132H cells when compared to the respective IDH1 wild-type cells (Fig. 2a, b). Moreover, treatment with the relatively selective Bcl-2 inhibitor ABT199 did not result in a difference in response among HCT116 IDH1-WT or HCT116 IDH1-R132H cells, emphasizing the predominant role of Bcl-xL in this context (Supplementary Fig. 1A). We next examined whether the synergistic anti-neoplastic activity of ABT263 and 2-HG can be attributed to selective inhibition of Bcl-xL. We, therefore, silenced U251 and LN229 glioblastoma cells for Bcl-xL. In both cell lines, knockdown of Bcl-xL resulted in a significant decrease in the fraction of viable cells among those cells treated in addition with 2-HG (Fig. 2c, d). In addition, treatment with the Bcl-2 inhibitor ABT199 did not result in a marked enhancement of the pro-apoptotic response in SF188, U251 and LN229 cells treated with ABT199/2-HG when compared to single-agent treatments (Supplementary Fig. 1B–D).

### Presence of an IDH1-mutated genotype and 2-HG sensitizes for BH-3 mimetic-mediated apoptosis

We next addressed the mechanism responsible for the enhanced anti-proliferative response toward ABT263 treatment in IDH1-mutated cells. Analysis of cell morphology lead to the observation that cells carrying the IDH1-R132H mutation developed typical features of apoptosis upon treatment with ABT263. In line with this finding, IDH1-mutated U87MG cells treated with ABT263 showed a significantly increased fraction of annexin V-positive cells (apoptotic cells) (Fig. 2e). Similarly, in IDH1-mutated T98G glioblastoma cells, PICPG-4 glioma cells (IDH1-R132H + , *PDGF* + , *TP53*−/−; derived from a transgenic mouse model) and isogenic HCT116 IDH1-R132H cells treatment with ABT263 resulted in an enhanced fraction of subG1 cells (apoptotic cells) when compared to T98G IDH1-WT, PPC2 (IDH1-WT) or HCT116 IDH1-WT cells (Fig. 1f and Supplementary Fig. 2A, B). On the molecular level, these findings were mirrored by enhanced cleavage of caspases 3, 9 and PARP in IDH1-mutated U87MG, T98G and HCT116 cells (Fig. 2g and Supplementary Fig. 2C, D). Microscopic analyses of glioblastoma cells treated with ABT263 in the presence of 2-HG revealed morphological changes typically associated with apoptosis (Fig. 1n). We, therefore, conducted flow cytometric analyses to elucidate whether the mechanism responsible for the anti-proliferative activity of a combined treatment with 2-HG and ABT263 involves the induction of apoptosis. As anticipated, treatment with ABT263 and 2-HG resulted in a synergistic increase in the fraction of annexin V-positive (apoptotic) U87MG, LN229, T98G, and SF188 glioblastoma cells and the fraction of subG1 cells in SF188 pediatric glioblastoma cells (Fig. 2h and Supplementary Fig. 3A–C). Consistent with this finding, LN229 and SF188 glioblastoma cells treated with ABT263 in the presence of 2-HG show a markedly enhanced cleavage of caspases 9, 3, and PARP, supporting the relevance of apoptosis as mechanism of the synergistic anti-cancer activity (Fig. 2j). To address whether the preferential pro-apoptotic response in IDH1-mutated cells toward ABT263 is at least in part mitochondrially driven, we performed staining for JC-1. IDH1-mutated U87MG glioblastoma cells showed a marked decrease in the mitochondrial membrane potential when subjected to treatment with increasing concentrations of ABT263 when compared to U87MG IDH1-WT cells (Fig. 2f). This finding supports at least a partial contribution of the mitochondrial pathway to the pro-apoptotic response. In support of this finding, treatment with the death receptor ligand TRAIL did not result in enhanced pro-apoptotic response in IDH1-mutant cells (Supplementary Fig. 4A–E). Furthermore, pro-apoptotic chemotherapeutics such as etoposide or paclitaxel did not display a varying response dependent on different IDH1 genotypes (Supplementary Fig. 4F, G).

Because ABT263 is a known inhibitor of the anti-apoptotic Bcl-2 family members Bcl-2 and Bcl-xL, we next examined whether the synergistic pro-apoptotic effect of the combination treatment would be at least in part be mitochondrially driven. We performed staining for JC-1 to detect changes in the mitochondrial membrane potential. As shown in Fig. 2i, treatment with ABT263 in the presence of 2-HG resulted in a marked increase in the fraction of LN229 glioblastoma cells undergoing a loss of the mitochondrial membrane potential.

### Mcl-1 is downregulated in IDH1-mutated glioblastoma cells and patient tumor samples

**Fig. 3:**
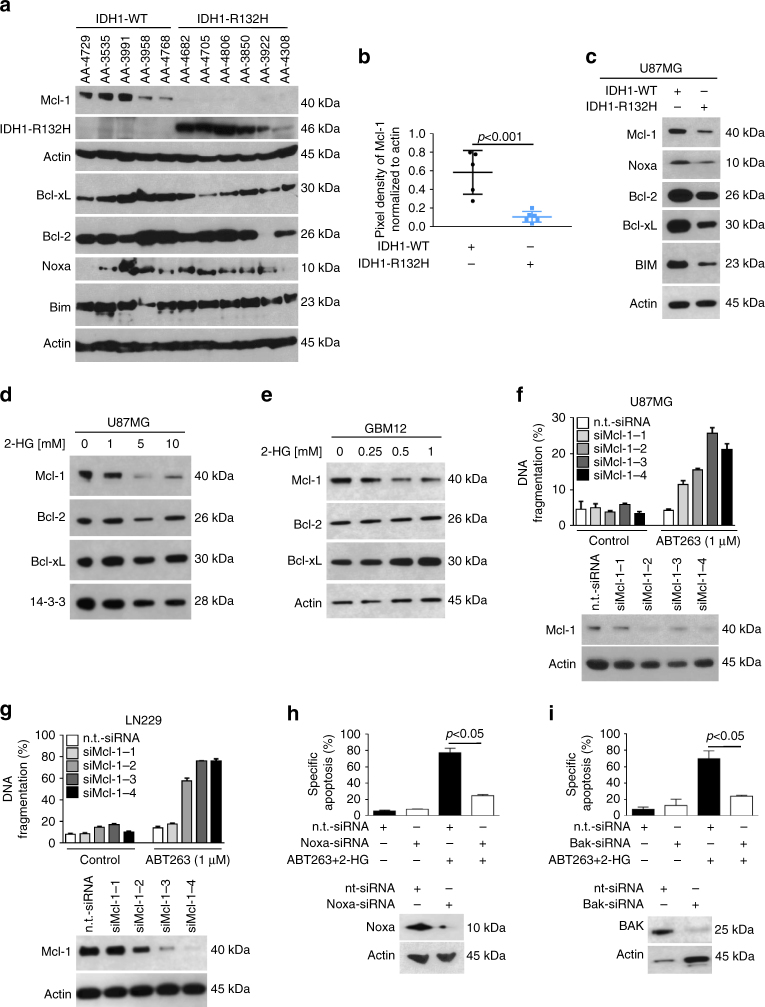
Mcl-1 is downregulated in IDH1-mutated gliomas. **a** Protein was extracted from human tumor samples of anaplastic astrocytoma expressing IDH1-WT or IDH1-R132H. Western blot analysis was performed for Mcl-1, Bcl-xL, Bcl-2, Noxa, and Bim. Actin served as loading control. **b** Scatter plot demonstrating Mcl-1 protein expression among IDH1-WT (*n* = 5) and IDH1-R132H (*n* = 6) expressing anaplastic astrocytoma samples. Densitometric analysis was performed using imageJ 1.47 v (http://imagej.nih.gov/ij). Pixel density was normalized to the respective Actin control. **c** Whole-cell extracts of U87MG IDH1-WT/IDH1-R132H cells were collected to determine base line protein expression of Mcl-1, Noxa, Bcl-2, Bcl-xL, and BIM by western blot analysis. Actin was used to ensure equal loading. **d**, **e** U87MG glioblastoma cells (**d**) and GBM12 patient-derived glioblastoma xenograft cells (**e**) were treated for 3 h (U87MG) or 24 h (GBM12) with increasing concentrations of 2-HG. Whole-cell extracts were collected prior to performing western blot analysis for Mcl-1, Bcl-2, and Bcl-xL. Western blot for Actin or 14-3-3 was performed to confirm equal loading. **f**, **g** U87MG (**f**) and LN229 (**g**) cells were treated with non-targeting (n.t.)-siRNA or 4 different Mcl-1-siRNAs and subjected to treatment with solvent or ABT263, for 24 h. Staining for propidium iodide was performed prior to flowcytometry. The fraction of subG1 cells was determined. Column: mean. *Error bar*: SD, *n* = 3. Western blot analysis was performed to confirm Mcl-1 knockdown. **h**, **i** Quantitative representation of LN229 cells treated with non-targeting (n.t.)-siRNA, Noxa-siRNA (**h**) or Bak-siRNA (**i**) prior to treatment with ABT263/2-HG or solvent. Staining for propidium iodide and flowcytometry were performed to determine the fraction of subG1 cells. Column: mean. *Error bar*: SD, *n* = 3. Whole-cell extracts were collected and western blot analysis was performed for Noxa and Bak to confirm appropriate knockdown

We next examined how the presence of the IDH1 mutation affects the expression of pro- and anti-apoptotic Bcl-2 family proteins. As shown in Fig. 3c and Supplementary Fig. 5A and B, U87MG and murine glioblastoma cells bearing the IDH1 mutation showed decreased expression of Mcl-1, Bcl-xL, and Bcl-2.

Compensatory upregulation of Mcl-1 is a well-defined mechanism of resistance toward ABT263. To extend our molecular studies to a more biologically relevant and disease-related paradigm, we determined basal Mcl-1 protein expression along with other anti- and pro-apoptotic Bcl-2 family members in tumor samples from patients with anaplastic astrocytoma stratified according to the presence or absence of the IDH1-R132H mutation. In IDH1-mutated tumor samples Mcl-1 expression was significantly decreased, suggesting a broad biological applicability of this observation (Fig. 3a, b). In contrast, no significant difference was found with respect to Bcl-2 or Bcl-xL expression among different IDH1 mutation status (Supplementary Fig. 5C).

To address the biological relevance of Mcl-1 as a driver for resistance toward ABT263, we performed knockdown experiments. U87MG and LN229 glioblastoma cells silenced for Mcl-1 by four different siRNAs showed a marked increase in the fraction of sub-G1 cells when treated with ABT263 (Fig. 3f, g).

Noxa and Bak are the main Mcl-1 interacting pro-apoptotic Bcl-2 family proteins. To elucidate whether Noxa is of importance for the synergistic pro-apoptotic effect of ABT263 and 2-HG, we performed knockdown experiments. Silencing Noxa results in a significant inhibition of ABT263/2-HG-mediated apoptosis (Fig. 3h and Supplementary Fig. 5D).

Mcl-1 sequesters the pro-apoptotic multi-domain effector protein Bak^4^. Once Noxa binds to Mcl-1, Bak is released^4^. Given that 1) the Noxa/Mcl-1 ratio is markedly increased upon treatment with AB263/2-HG and 2) Noxa knockdown results in an attenuation of the pro-apoptotic response, one would expect that knockdown of Bak also attenuates ABT263/2-HG-mediated apoptosis. As anticipated, selective knockdown of Bak resulted in a significant reduction of apoptosis in cells treated with ABT263 and 2-HG (Fig. 3i and Supplementary Fig. 5E).

Based on our observation that in IDH1-mutated glioblastoma cells Mcl-1 expression is decreased, we next examined whether 2-HG treatment would recapitulate this molecular feature. Both U87MG and GBM12 cells displayed a concentration-dependent decrease in Mcl-1 expression when treated with 2-HG for 24 h (Fig. 3d, e). To further assess the subjacent mechanism, we performed real-time rtPCR experiments. Mcl-1 messenger RNA (mRNA) expression was not decreased but even increased in U87MG cells suggesting a post-transcriptional mechanism (Supplementary Fig. 5F).

### Mutant IDH1 R132H and 2-HG blunt oxidative energy metabolism

**Fig. 4:**
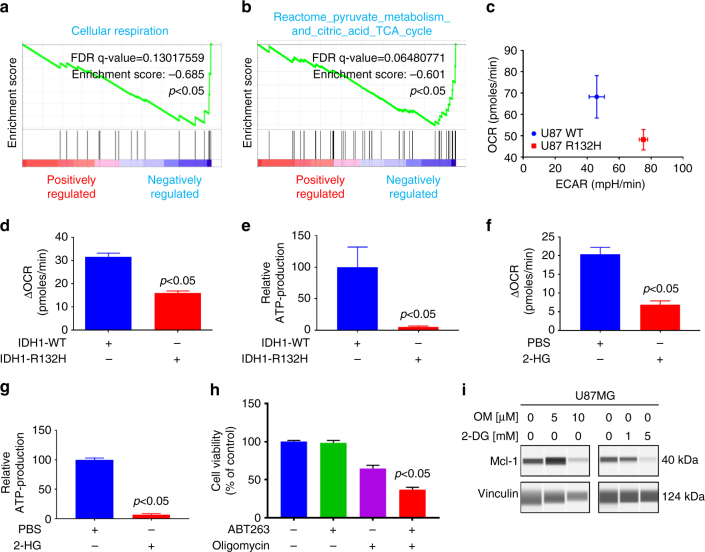
U87-mutated IDH1 R132H status and the onco-metabolite 2-HG impair oxidative mitochondrial metabolism, suppressing ATP and rendering cells vulnerable to Bcl-xL inhibition, **a**, **b** U87 wild-type and IDH1 R132H-mutated glioblastoma cells were submitted for transcriptome analysis and subsequently analyzed by GSEA. Shown are GSEA plots with the respective statistical analysis. These results suggest an impairment of oxidative metabolism with downregulation of *SDHD*, *IDH3A*, *PDHX*, and *DLAT*. **c** Wild-type or IDH1 R132H-mutated glioblastoma cells were analyzed for oxygen consumption rate (*OCR*) or extracellular acidification rate (ECAR) by Seahorse XFp Flux analyzer (metabolic phenotype analysis). Column: mean. *Error bar*: SD, *n* = 3. **d** Wild-type and IDH1 R132H-mutated glioblastoma cells were analyzed for mitochondrial oxygen consumption rate. Column: mean. *Error bar*: SD, *n* = 3. **e** Wild-type and IDH1 R132H-mutated glioblastoma cells were analyzed for relative oxidative phosphorylation mediated ATP production. Column: mean. *Error bar*: SD *n* = 3. **f**, **g** U87 cells were treated with vehicle or 1 mM 2-HG and analyzed as in **e**, **f**. Column: mean. *Error bar*: SD, *n* = 3. **h** U87 cells were treated with 1 μM ABT263, 5 μM Oligomycin or the combination of both. Subsequently, cells were analyzed by CellTiter-Glo® assay. Column: mean. *Error bar*: SD, *n* = 3. **i** U87 cells were treated with 2-DG or oligomycin with the indicated concentrations and analyzed by capillary electrophoresis for the expression of Mcl-1 and Vinculin, using the Wes instrument. Student’s *t*-test or one-way ANOVA with post-hoc Tukey analysis were used for statistical analysis and a *p*-value of <0.05 was considered statistically significant

To determine the underlying mechanism of reduced Mcl-1 protein levels in IDH1 mutant cells, we conducted transcriptome analysis with subsequent gene set enrichment analysis. We found that U87 IDH1-mutated glioblastoma cells displayed a reduction in genes related to oxidative metabolism, including cellular respiration, tricarboxylic acid (TCA) cycle, and pyruvate metabolism (Fig. 4a, b an﻿d Su﻿pplementary ﻿Fig. 6A)). To address this finding, we analyzed the basal oxygen consumption rate (OCR) and extracellular acidification rate (ECAR) in wild-type and mutant IDH1 cells. (Fig. 4c). Based on these findings, we analyzed the metabolic phenotype of IDH1 wild-type and IDH1 R132H-mutated glioblastoma cells. Our findings suggest that the IDH1 mutation leads to a metabolic shift with enhanced glycolysis and reduced oxidative phosphorylation (Fig. 4c), supporting the findings observed in the transcriptome analysis. In agreement, IDH1 R132H-mutated or 2-HG-treated glioblastoma cells showed less mitochondrial respiration and significant reduction in ATP production (Fig. 4d–g). As early as one hour after treatment with 1 mM 2-HG, glioblastoma cells had a significant reduction in baseline respiration, maximum respiratory capacity, spare capacity, and ATP production (Supplementary Fig. 7A–C). These findings suggest that 2-HG has a direct impact on mitochondrial respiration (Supplementary Fig. 7). Consistently, total ATP levels were also reduced in 2-HG-treated and IDH1 R132H-mutated cells (Supplementary Fig. 6B). To test the hypothesis that ATP depleting reagents are capable of enhancing ABT263-mediated cell death, glioblastoma cells were treated with two bona-fide ATP reducing compounds, Oligomycin and 2-DG. In the presence of ABT263, both molecules dramatically enhanced the reduction of cellular viability as compared to each compound on its own (Fig. 4h and Supplementary Fig. 6C). Since ATP levels affect cellular survival, we determined as to whether or not Oligomycin and 2-DG are capable of reducing Mcl-1 protein levels. Our findings suggest that Oligomycin and 2-DG suppress Mcl-1 protein levels (Fig. 4i), explaining in part why ABT263 synergizes with these two compounds.

### Mutated IDH1 and 2-HG lead to a reduction of protein synthesis and mTORC1 signaling

**Fig. 5:**
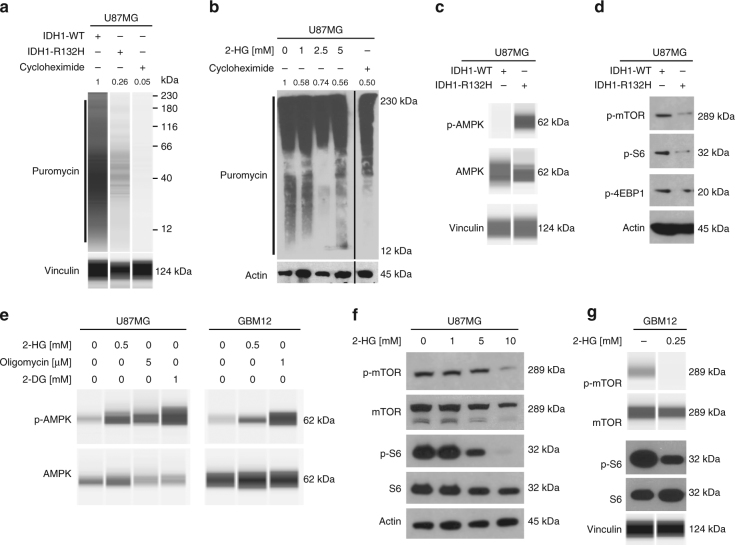
Impaired protein synthesis leads to downregulation of Mcl-1 in IDH1-mutated glioblastomas. **a** U87MG IDH1-WT/IDH1-R132H cells were pretreated with puromycin. Whole-cell extracts were collected and capillary electrophoresis was performed for puromycin. Vinculin served as loading control. Treatment with 10 μg ml^−1^ cycloheximide served as a negative control. Densitometric analysis was performed using imageJ 1.47 v (http://imagej.nih.gov/ij). Pixel density was normalized first to the respective Vinculin and second to U87MG IDH1-WT cells. **b** U87MG cells were treated for 24 h with increasing concentrations of 2-HG prior to incubation for 2 h with puromycin (10 μM). Treatment with the protein synthesis inhibitor cycloheximide (10 μg ml^−1^) served as a negative control. Whole-cell extracts were collected prior to western blot analysis for puromycin. Densitometric analysis was performed using imageJ 1.47 v (http://imagej.nih.gov/ij). Pixel density was determined and normalized first to the respective Actin signal and second to U87MG cells treated with solvent. **c** Whole-cell extracts of U87MG IDH1-WT/IDH1-R132H cells were collected prior to capillary electrophoresis for p-AMPK (Threonine 172) and AMPK. Vinculin served to control for equal loading. **d** Whole-cell extracts of U87MG IDH1-WT/IDH1-R132H cells were collected prior to western blot analysis for p-mTOR, p-S6, and p-4EBP1. Western blot for Actin was performed to confirm equal loading. **e** U87MG and GBM12 cells were treated for 48 h with 2-HG, oligomycin or 2-DG as indicated. Whole-cell extracts were collected prior to capillary electrophoresis for pAMPK and AMPK. **f**, **g** U87MG (**f**) and GBM12 (**g**) cells were treated with 2-HG as indicated. Whole-cell extracts were collected. Western blot analysis or capillary electrophoresis was performed for p-mTOR, mTOR, p-S6 and S6. Either Actin or Vinculin served as loading control

Reduction in ATP-levels is associated with cellular stress and a subsequent reduction in protein synthesis. To further evaluate this hypothesis, we performed sunset assays^5^. As shown in Fig. 5a, U87MG IDH1-R132H cells treated with puromycin show a significantly decreased puromycin labeling when compared to IDH1-WT cells confirming decreased protein synthesis.

Moreover, U87MG cells treated with increasing concentrations of 2-HG show a marked decrease in puromycin signal, which indicates a decrease in translational activity (Fig. 5b).

Mammalian target of rapamycin (mTOR) signaling represents an important regulator of cellular proliferation in cancer. To link mTOR signaling with low energy levels, we analyzed the phosphorylation status of AMPK. IDH1-mutated U87MG glioblastoma and NCH644 glioma stem-like cells display significantly enhanced phosphorylation of AMPK (Fig. 5c and Supplementary Fig. 6D). To confirm whether mTOR signaling is affected in IDH1-mutated glioblastoma cells, we performed western blot analyses for p-mTOR and its downstream effector p-S6. In IDH1-mutated U87MG cells, mTOR signaling was significantly downregulated (Fig. 5d). Moreover, phosphorylation of 4EBP1 was found to be decreased. Furthermore, we found that glioblastoma cells treated with, 2-HG, Oligomycin, and 2-DG showed an increase of phosphorylation of AMPK (Fig. 5e), suggesting lower levels of ATP. In turn, active AMPK blunts the activity of mTORC1.

Consequently, in U87MG and GBM12 cells, treatment with 2-HG resulted in a significant decrease in p-mTOR and p-S6 expression (Fig. 5f, g, Supplementary Fig. 8A).

To further address whether alterations in proteasomal degradation of Mcl-1 add to the post-translational mechanism we treated U87MG cells with 2-HG in the presence or absence of the proteasome inhibitor MG132. Combined treatment with 2-HG and MG132 leads to a rescue of Mcl-1 levels, indicating that the 2-HG-mediated decrease in Mcl-1 is at least in part mediated by enhanced proteasomal degradation (Supplementary Fig. 8B). Finally, Mcl-1 mRNA levels did not decrease in the presence of 2-HG (Supplementary Fig. 5F), confirming a predominantly post-transcriptional regulation of Mcl-1.

### ABT263 prolongs survival in an orthotopic IDH1-mutated glioblastoma xenograft model

**Fig. 6:**
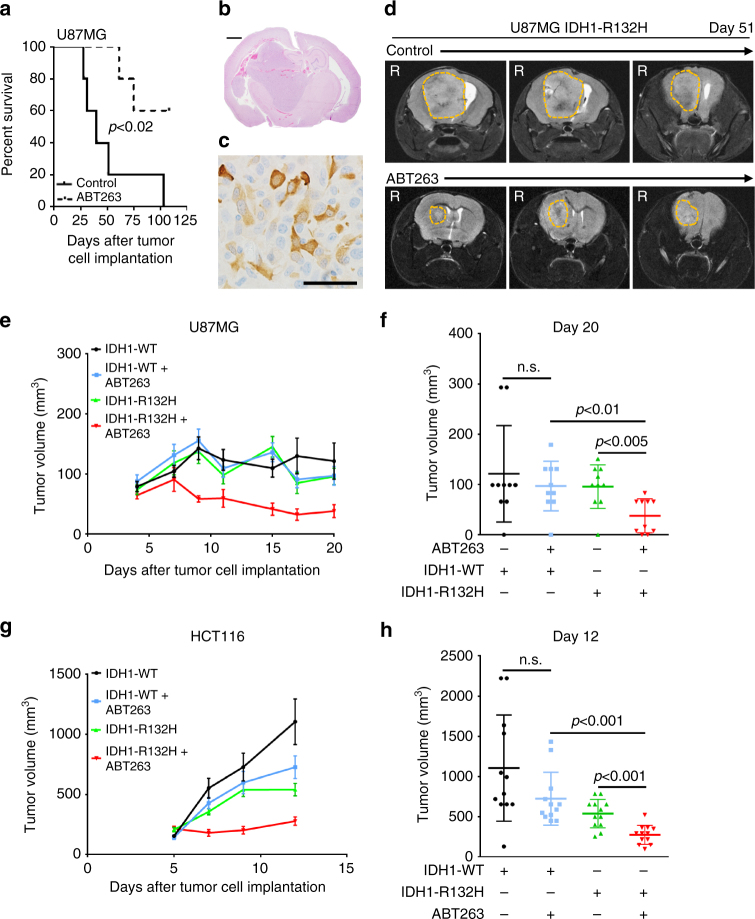
ABT263 extends overall survival in an orthotopic glioblastoma model of IDH1-mutated U87MG. **a** U87MG IDH1-R132H cells were implanted into the right striatum of Nude mice. After establishment of tumors, two groups were formed and animals were treated with either vehicle (*n* = 5; median survival = 40 days) or three times per week with 100 mg kg^−1^ ABT263 (*n* = 5; median survival > 108 days). Kaplan–Meier curves were generated for survival analysis. Comparison of survival curves was performed using Log-rank (Mantel-Cox) test. **b** Representative histological brain section from a vehicle-treated animal stained with H&E. *Scale bar*: 1 mm. **c** Immunohistochemical staining for IDH1-R132H in a tumor derived from U87MG IDH1-R132H cells. Magnification, 40×. *Scale bar*: 50 µm. **d** Representative MRI images of vehicle treated and ABT263-treated animals carrying U87MG IDH1-R132H tumors. **e** 1 × 10^6^ U87MG IDH1-WT or IDH1-R132H cells were implanted subcutaneously. After tumor formation animals were randomized into two groups per IHD1 status (*n* = 10 tumors per group) and treated intraperitoneally with vehicle or ABT263 100 mg kg^−1^ 3× per week. **f** Scatter plot indicating the tumor size of animals treated as described for E on day 20 after tumor cell implantation. **g** In all, 1 × 10^6^ HCT116 IDH1-WT or IDH1-R132H colorectal cancer cells were implanted subcutaneously. After tumor formation animals were randomized into two groups per IDH1 status (*n* = 12 tumors per group) and treated on 5 consecutive days intraperitoneally with vehicle or ABT263 75 mg kg^−1^. **h** Scatter plot indicating the tumor size of animals treated as described for **h** on day 12 after tumor cell implantation. **e**–**h** Data are presented as mean and SEM **e**, **g**/SD, **f**, **h**). Student’s *t*-test was used for statistical analysis and a *p*-value of <0.05 was considered statistically significant

Our in vitro data showed an enhanced anti-neoplastic activity of ABT263 in IDH1-mutated glioblastoma cells. We next examined whether this finding also translates into enhanced therapeutic efficacy in vivo. In a heterotopic subcutaneous glioblastoma model, animals with U87MG IDH1-R132H tumors subjected to treatment with ABT263 had a significantly decreased tumor growth rate and even showed tumor regression when compared to vehicle treatment (Fig. 6e, f). In contrast, no significant difference in tumor growth was noted between vehicle and ABT263-treated animals with U87MG IDH1-WT tumors. Given that there was no difference between non-treated and ABT263 IDH1 wild-type U87MG skin xenograft tumors, we focused our intracranial studies on the IDH1-R132H tumor cells. To this end, U87MG IDH1-R132H glioblastoma cells were implanted intracranially and allowed to form tumors. Once tumors formed, animals were randomized to treatment with either vehicle or ABT263. In animals with tumors carrying the IDH1-mutation, treatment with ABT263 resulted in a significantly prolonged overall survival (Fig. 6a–d). After 103 days, all control animals were dead, while three ABT263-treated animals (out of five) were still alive (median survival vehicle animals: 40 days; median survival ABT263-treated animals: > 103 days; *p* < 0.02).

We next assessed whether ABT263 also yields a stronger anti-cancer activity in an IDH1 R132H-expressing isogenic tumor model. For that purpose, IDH1 WT or IDH1 R132H HCT116 cells were implanted subcutaneously into nude mice. As shown in Fig. 6g, h, treatment with ABT263 resulted in a marked reduction of the tumor growth rate of both HCT116 IDH1 WT- as well as HCT116 IDH1 R132H-expressing tumors. However, toward the end of the study, the mean size of tumors expressing the IDH1 mutation in animals treated with ABT263 was significantly smaller when compared to tumors from vehicle-treated animals. In contrast, the mean size of tumors expressing the wild-type form of IDH1 did not significantly differ between tumors from vehicle-treated and ABT263-treated animals.

**Fig. 7:**
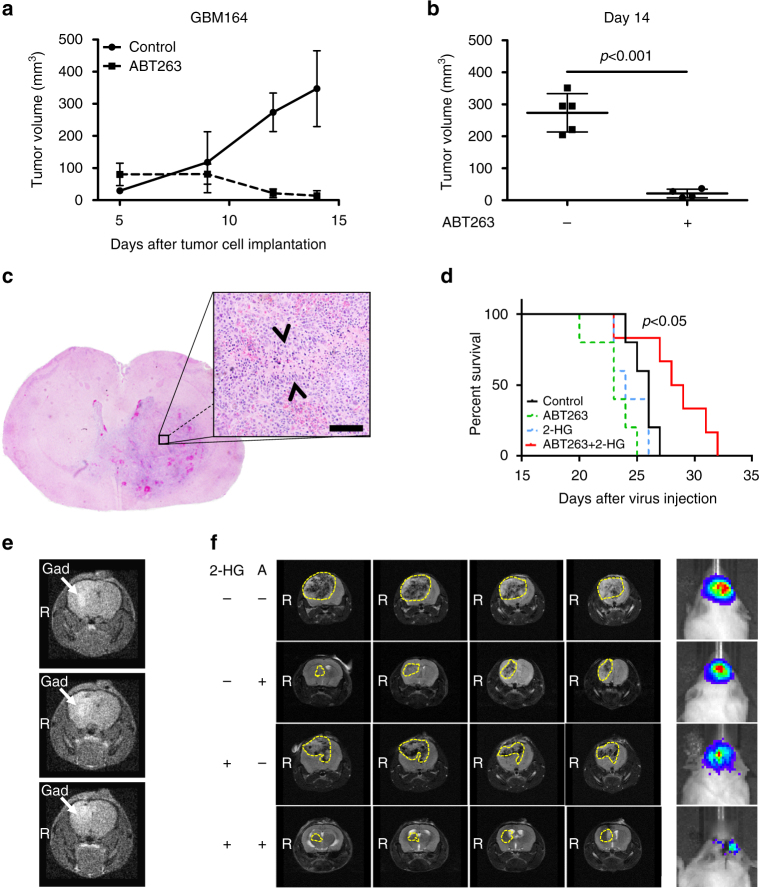
Treatment with ABT263 in combination with 2-HG prolongs survival in a mouse model generated by orthotopic injection of PDGF-IRES-Cre retrovirus. **a** GBM164 cryomush was implanted subcutaneously. After tumor formation, animals were randomized into two groups (*n* = 4–5 tumors per group) and treated intraperitoneally with vehicle or ABT263 75 mg kg^−1^ three times a week. **b** Scatter plot indicating the tumor size of animals treated as described for **a** on day 14 after tumor cell implantation. **a**, **b** Data are presented as mean and SD. **c** Representative microphotograph of a tumor bearing brain from a mouse treated with vehicle (H&E, 2× magnification) and microphotograph at higher magnification. *Arrow heads* indicate the formation of pseudopalisading necrosis. *Scale bar*: 50 µm. **d** Kaplan–Meier curves of animals treated with vehicle (*n* = 5; median survival = 26 days), ABT263 intraperitoneally 100 mg kg^−1^ 3× per week (*n* = 5; median survival = 23 days), 10 mM 2-HG by convection-enhanced delivery for 7 days (*n* = 5; median survival = 24 days) or ABT263 and 2-HG combined (*n* = 6; median survival = 28.5 days). Statistical comparison of survival curves was performed using Log-rank (Mantel-Cox) test. **e** Sequential representative brain MRI scans showing the intracerebral accumulation of Gadolinium after convection-enhanced delivery (Bruker ICON^TM^, 1 Tesla MR imager). **f** Sequential representative brain MRI scans of animals treated as indicated and described in **d** using a Bruker BioSpec^TM^, 9.4 Tesla MR imager. Interrupted *yellow line* marks the tumor outline. Representative photographs visualizing the bioluminescent signal emitted by formed tumors after intraperitoneal injection of 150 mg kg^−1^ d-Luciferin (GOLD Biotechnology, St Louis, MO) using an IVIS Spectrum optical imaging system (Perkin Elmer, Waltham, MA)

Next, we tested the efficacy of ABT263 in a patient-derived xenograft model system of IDH mutant glioblastoma (GBM164). After two weeks of treatment, Bcl-xL inhibition led to a significant tumor regression (*p* < 0.001) (Fig. 7a, b).

### Treatment with ABT263 results in prolonged survival in the presence of 2-HG in vivo

To assess whether treatment with ABT263 in the presence of 2-HG provides a survival benefit in vivo, we used an orthotopic model of proneural glioblastoma^6, 7^. Intracranial tumors (*PDGF* + , *PTEN* ^−/−^, *p53* ^−/−^, luciferase + ) were induced through retroviral injection and tumor formation was verified by IVIS imaging (Fig. 7c–f). Convection-enhanced delivery was used for local application of 2-HG in order to mimic its local distribution as seen in IDH1-mutant tumors. Therefore, 2 weeks after injection of the virus micro-osmotic pumps were placed to infuse 2-HG or vehicle intracranially for 7 days accompanied by intra-peritoneal treatment with ABT263 or vehicle. Delivery of 2-HG was verified by detecting Gadolinium signal in magnetic resonance imaging (MRI) scans (Fig. 7e). As shown in Fig. 7d, treatment with ABT263 provided a statistically significant survival benefit in the presence of 2-HG. This observation was accompanied by a smaller tumor size and a weaker luminescence signal (Fig. 7f).

## Discussion

In low-grade gliomas and IDH-mutant glioblastomas, mutations in IDH1 and less commonly in IDH2 predict significantly improved overall survival when compared to wild-type IDH glioblastomas. While IDH1 is localized in the cytoplasm, IDH2 resides in the mitochondria^8^. In contrast to IDH3, which is localized to the mitochondria, utilizes NADH_2_ and is reversible, both IDH1 and IDH2 catalyze a reaction that is irreversible, which leads to the generation of NADPH_2_ and alpha-ketoglutarate. In turn, NADPH_2_ may aid in entertaining an anabolic state of cancer cells by providing electrons for certain steps in metabolic synthesis, such as fatty acid and nucleotide synthesis (ribonucleotide reductase). In IDH1-mutated tumors, NADPH_2_ is a co-factor for the reduction of alpha-ketoglutarate to 2-HG. 2-HG accumulates to significant levels in IDH-mutated glioblastomas, reaching up to 30 mM. Due to its structural similarity to alpha-ketoglutarate 2-HG interferes with the normal function of dioxygenases that require alpha-ketoglutarate as a co-factor^9^. These dioxygenases are especially relevant in histone and DNA demethylation, which ultimately leads to a hypermethylated phenotype in IDH1-mutated neoplasms^10^. Given its abundant presence in IDH1-mutated tumors, 2-HG is being evaluated as a biomarker in imaging studies, serum and urine of patients, which allows monitoring disease progression. Given all these features of 2-HG, it was tempting to find inhibitors that block the neo-enzymatic activity of mutated IDH enzymes. One of these inhibitors is AGI-5198, which in model systems of IDH1-mutated glioblastomas caused a significant reduction in the levels of 2-HG^11^. Other groups have taken similar approaches in other tumor entities as well^12^.

In this study, we utilized the concept of synthetic lethality to efficiently target glioblastomas bearing the IDH1 mutation. This strategy might be a welcome contribution for the treatment of IDH-mutated neoplasms for several reasons, including the notion that IDH inhibitors might not be efficient in every patient with an IDH mutation and the concern of targeting a mutated enzyme that confers a better prognosis. Concerning immunotherapy, such approaches might only be feasible for IDH1-mutated low-grade gliomas, but not suitable for high-grade tumors. Despite the fact that glioblastomas are heterogeneous, the IDH1 mutation is uniformly present within a mutated tumor^13^, suggesting that “synthetic lethality”-based treatment approaches should affect the majority of cancer cells within IDH-mutated tumors. Our findings suggest that in solid tumors, such as glioblastomas, the inhibition of Bcl-xL elicits synthetic lethality in IDH1-mutated neoplasms. We utilized several experimental approaches to verify this observation, including patient-derived IDH1-mutated sphere cultures, isogenic cell cultures and cells that were transfected or transduced with either wild-type IDH1 or mutated IDH1 R132H. This plethora of model systems is necessary since isogenic cells might not have the classical genetic background in which IDH1 mutation usually arise. All model system unequivocally confirmed that IDH1 R132H renders tumor cells more prone to the effects of inhibition of Bcl-xL either by siRNA or pharmacologically through the BH3-mimetic, ABT263. It is interesting to note that the “specific” Bcl-2 inhibitor, ABT199, did not mediate synthetic lethality in IDH1-mutated cells. Similar results were seen with knockdown experiments, involving Bcl-2 alone. Our findings extend previous studies of glioblastomas indicating a special role for mutated IDH1 to modulate cell death pathways, such as autophagy and apoptosis^8, 14, 15^. Our mechanistic findings are in contrast to an earlier report, which suggested that in non-solid tumors (myeloid leukemia) Bcl-2 antagonism is synthetically lethal with mutated IDH1 or IDH2^16^. Related research has indicated that solid tumors are generally more dependent on Bcl-xL than on Bcl-2, which is in agreement with our observations^17^. The disadvantage of the inhibition of Bcl-xL is the fact that platelets are dependent on Bcl-xL and extensive inhibition of Bcl-xL may, therefore, lead to thrombocytopenia^17^. However, in patients that are predicted to be sensitive to Bcl-xL inhibition, low platelet counts may not be a concern since tumor cells due to a genetic alteration may be more prone to Bcl-xL inhibition than platelets.

To elucidate the mechanisms by which IDH1 R132H-mutated cells are more prone to Bcl-xL inhibition, we hypothesized that these effects might be mediated by the oncometabolite, 2-HG^18^. Consistent with this postulate are our findings that IDH1-mutated glioblastomas and our engineered IDH1-mutated glioblastoma cells displayed lower levels of Mcl-1 and treatment with a cell-permeable form of 2-HG (pharmacologically relevant concentrations) suppressed Mcl-1 protein levels in IDH1 wild-type cells. Mechanistically, 2-HG-mediated reduction of Mcl-1 protein levels is explained by less protein synthesis and a partial shutdown of mTORC1 signaling^19^, which is in keeping with a recent report demonstrating that 2-HG prolongs the lifespan of *C. elegans* in part by inhibition of mTORC1 signaling^3^. While there are multiple possibilities of suppression of mTOR signaling, 2-HG appears to interfere with oxidative phosphorylation at the level of the ATP-synthase, culminating in a state of energy depletion and suppression of mTORC1 signaling^3^. Our present findings support those earlier observations since in our model systems mutant IDH1 leads to a metabolic reprogramming, with a more “glycolytic” phenotype rather than “oxidative”. As a result, both mutant IDH1 and 2-HG-treated cells displayed lower baseline OCRs and ATP levels, which in part mediated a reduction of protein synthesis, mTORC1 signaling and finally a decline in Mcl-1. The precise mechanism as to how mutant IDH1 cells become more glycolytic is likely to involve multiple factors. In addition to the direct impact of 2-HG on cellular respiration, effects on other important glycolytic regulatory enzymes, such as pyruvate dehydrogenase, which is at the interface between oxidative and purely “aerobic glycolytic” metabolism and is regulated by a complex cascade of phosphorylation, require further analysis. Aside from its regulation on protein synthesis, we also found that 2-HG impacts the stability of Mcl-1 through modulation of its proteasomal turnover. This finding is consistent with numerous other reports that show that various compounds regulate the proteasomal degradation of Mcl-1^20–23^ and confirms the notion that Mcl-1 is commonly regulated at the posttranslational level.

Our results differ mechanistically from earlier findings in myeloid leukemia^16^, which did not display changes in Mcl-1 and Noxa levels in cells treated with 2-HG. However, these observations might be cell- or tumor-type specific. For instance, hematological malignancies might respond in a different manner compared to solid malignancies, such as glioblastomas or cholangiocarcinomas. We also verified by siRNA studies that Noxa is involved in the cell death induced by Bcl-2/Bcl-xL inhibition and 2-HG exposure, suggesting that Noxa is required in the death and not a “bystander” effect. In agreement with a Mcl-1/Noxa-dependent effect is our finding that 2-HG in combination with ABT263 elicits Bak-dependent cell death since Bak is known to primarily bind to Mcl-1, while Bax is incapable of interacting with Mcl-1.

Finally, we demonstrated that Bcl-xL inhibition is highly efficacious in several in vivo model systems of mutated IDH1, including an orthotopic xenograft model of glioblastoma and a patient-derived xenograft. These results should be seen in context of recent observations published by others. For instance, the Lai group demonstrated that IDH1 mutations render glioblastoma cells more sensitive to radiation^24^. Interference with glutamine metabolism or NAD + depletion led to a more pronounced growth reduction in IDH1-mutated cells^25, 26^. In connection with DNA-damaging agents, it was shown that IDH1-mutated glioblastomas are more sensitive to a certain class of DNA alkylating reagents^27^. Src inhibition causes synthetic lethality in IDH-mutated cholangiocarcinomas. Similarly, the c-myc and BRD4 inhibitor, JQ1, was shown to be efficacious in IDH1-mutated cells^28^. In total, these data suggest that induction of synthetic lethality in IDH-mutated neoplasms is feasible and potentially highly efficacious.

In summary, our work suggests that glioblastomas harboring an IDH1 mutation might benefit from the addition of a compound that efficiently interferes with Bcl-xL.

## Methods

### Ethics statement

All procedures were in accordance with Animal Welfare Regulations and approved by the Institutional Animal Care and Use Committee at the Columbia University Medical Center. For studies involving human samples Columbia IRB approved these studies. For human studies, informed written consent was obtained from all patients.

### Reagents

ABT263 and ABT199 were purchased from Selleckchem (Houston, TX). Compounds were dissolved in dimethyl sulfoxide (DMSO) and 10 mM stock solutions were prepared and stored at −20 °C. (2 R)-2-hydroxyglutaric acid octyl ester sodium salt was purchased from Cayman Chemical Company (Ann Arbor, MI). A 50 mM stock solution was prepared in phosphate-buffered saline and stored at −20 °C.

### Cell cultures and growth conditions

BT-142, U87MG, T98G and LN229 human glioblastoma cell lines were obtained from the American Type Culture Collection (Manassas, VA) and were negatively tested for Mycoplasma. NCH612 and NCH644 stem cell-like glioma cells were obtained from Cell Line Services (CLS, Heidelberg, Germany) and negatively tested for Mycoplasma. HCT116 IDH1 parental (clone: 326) and HCT116 IDH1 (R132H/ + ) (clone: 97) human colorectal cancer cells were purchased from Horizon Discovery (Cambridge, MA) and negatively tested for mycoplasma. The identities of the cell lines we purchased were confirmed by the respective source of purchase. SF188 pediatric glioblastoma cells were kindly provided by Dr Craig Thompson (Memorial Sloan Kettering Cancer Center, New York, NY). MGPP-3 (PDGF + , p53(−/−), PTEN (−/−)) are murine proneural glioblastoma cells, which were generated by Dr Peter Canoll (Columbia University, New York, NY). U251 glioblastoma cells were kindly provided by Dr James Goldman (Columbia University, New York, NY). GBM12 and GBM164 glioblastoma cells were kindly provided by Dr J. Sarkaria (Mayo Clinic, Rochester, MI). All cells were cultured as previously described^29, 30^. T98G, LN229, U251, and MGPP-3 cells were cultured in Dulbecco's Modified Eagle's Medium (DMEM) with 10% fetal bovine serum (FBS), 4.5 g l^−1^ glucose, 4mM L-glutamine, 1 mM pyruvate and 5 μg ml^−1^ Plasmocin^TM^ for maintenance. For experimental conditions these cells were cultured in DMEM containing only 1.5% FBS to mimic the nutrition starved setting in tumors. For the culture of SF188 the fore-mentioned medium was supplemented in addition with 2mM l-alanyl-l-glutamine (GlutaMAX^TM^-I, Gibco, Japan). NCH644 and NCH612 were cultured in MG-43 medium (CLS, Heidelberg, Germany) for both maintenance and experiments.

### Vector constructs and viral transduction

For the generation of retroviral particles for IDH1 R132H and IDH1 wild-type we utilized the following plasmids: pLPCX-IDH1 and pLPCX-IDH1 R132H. These plasmids were kindly provided by Dr Albert Lai (David Geffen School of Medicine at UCLA, Los Angeles, CA)^24^. In addition, we utilized lentiviral doxycycline-inducible pTRIPZ IDH1 R132H and pTRIPZ IDH1wild-type vectors, which were kindly provided by Dr Ravindra Majeti (Stanford University School of Medicine, Stanford, CA)^16^.

### Cell viability assays

In order to examine cellular proliferation, 3-[4, 5-dimethylthiazol-2-yl]-2, 5-diphenyltetrazolium bromide (MTT) or CellTiter-Glo^®^ assays were performed as previously described^31–33^.

### Measurement of apoptosis and mitochondrial membrane potential

For annexin V staining the FITC Annexin V Apoptosis Detection Kit I (BD Pharmingen, USA) was used according to the manufacturer’s instructions. Staining for propidium iodide or JC-1 was performed as previously described^31^. The data were analyzed with the FlowJo software (version 8.7.1; Tree Star, Ashland, OR).

### Protein extraction from human brain tumor samples

Human tumor samples of human anaplastic astrocytomas were kindly provided by Dr Peter Canoll (Columbia University, New York, NY). To extract protein, 100–200 mg of tumor tissue was mechanically dissociated in RIPA buffer. After incubation on ice for 15 min, samples were centrifuged at 14,000×*g* for 10 min. The supernatant was collected and sonicated 3× for 5 s each on ice prior to subsequent western blot analyses.

### Western blot analysis

Specific protein expression in cell lines was determined by western blot analysis as described before^33, 34^ using the following primary antibodies: Mcl-1 (1:500; #5453 CST: Cell Signaling Technology, Danvers, MA), Bcl-2 (1:500; #4223 CST), mutation specific IDH1 R132H antibody (1:250; #DIA-H09 Dianova GmbH, Hamburg, Germany), human caspase-9 (1:1,000; #9502 CST), cleaved caspase-3 (1:250; #9664 CST), total PARP (1:1000; #9532 CST), cleaved PARP (Asp214, 1:1000; #9541 CST), Bcl-xL (1:500; #2764 CST), Bim (1:500; #2933 CST), Bak (1:500; #6947 CST) Noxa (1:500, clone 114C307; Calbiochem), β-actin (1:8,000, clone AC15; A1978 Sigma Aldrich), Vinculin (1:1000, #ab129002 Abcam). Secondary HRP-linked antibodies were purchased from Santa Cruz Biotechnology.

Capillary electrophoresis was performed on the Wes simple instrument (Protein simple, San Jose, CA) using the 12–230 kDa Wes separation module and anti-rabbit or anti-mouse detection modules according to the manufacturer’s instructions. Antibody concentrations are available on the Protein simple website. Uncropped western blot and capillary electrophoresis data are shown in Supplementary Fig. 9.

### Metabolic flux analysis

For the determination of OCR and ECAR, the Seahorse XFp Flux analyzer was utilized in accordance with the manufacturer instructions and published protocols for the indicated cell lines.

### Sunset assay

To determine effects on protein synthesis a non-radioactive puromycin-based assay was used^5^. For this assay, cells were treated for 2 h with puromycin at a concentration of 10 μg ml^−1^. Afterwards, whole-cell extracts were harvested and western blot analysis was performed using anti-puromycin (1:10,000, clone 12D10; EMD Millipore). Cells treated with cycloheximide at a concentration of 10 μg ml^−1^ served as negative control.

### Transfections of siRNAs

Non-targeting siRNA-pool (ON-TARGETplus Non-targeting Pool, # D-001810-10-05) and Mcl-1 (SMARTpool: ON-TARGETplus Mcl-1 siRNA, L-004501-00-0005) were purchased from Dharmacon and transfected as previously described^21, 35^. For single siRNA experiments involving Mcl-1, the following sequences were employed: siRNA-1: GGU UUG GCA UAU CUA AUA A; siRNA-2: GAA GGU GGC AUC AGG AAU G, siRNA-3: GAU UAU CUC UCU CGG UAC CUU, siRNA-4: CGA AGG AAG UAU CGA AUU U (Dharmacon). For siRNA experiments, involving Bcl-xL, siRNAs were either used from Ambion (Silencer^®^ Select s1920; AUA CUU UUG UGG AAC UCU ATT) or Cell Signaling Technology^®^ (Signal silence^®^ Bcl-xL siRNA I; #6362). Silencing of PMAIP1 was performed using Silencer^®^ Select siRNA-1 (s10708; AGA UAU GAA UGU UUC UAA ATT) and siRNA-2 (s10709; AGU CGA GUG UGC UAC UCA ATT) from Ambion. BAK1 knock-down was performed using Silencer^®^ Select s1880 from Ambion. Briefly, cells were incubated for 6 h with the formed complexes of Oligofectamine^®^ 2000 (Invitrogen, Carlsbad, CA) and the respective siRNA (12-well condition) in DMEM without FBS and antibiotics. After 6 h, FBS was added to a total concentration of 1.5%.

### Microarray and GSEA analysis

Affymetrix Human Gene 2.0 ST microarrays were utilized to assess the transcriptome for U87MG IDH1 wild-type vs. IDH1 R132H. To obtain gene-level expression values, the affy package (version 1.36.1) was utilized. The genes on the array were assigned their respective Entrez Gene identifiers. The log2-transformed fold changes between the different groups (IDH1 wild-type vs. IDH1 mutant) were ranked. The rank-lists served as the source for gene set enrichment analysis (GSEA, version 2.2.1), utilizing gene sets available from the Molecular Signatures Database, version 5.0^36, 37^.

### Real-time rtPCR and cDNA synthesis

Real-time rtPCR was performed as described before^38^ using the primers as outlined in Supplementary Table 3.

### Subcutaneous xenograft model

In all, 1 × 10^6^ U87MG/HCT116 IDH1-WT/IDH1-R132H cells suspended in Matrigel (1:1) or IDH1-mutant GBM164 cryomush were implanted subcutaneously into the flanks of 6–8-week-old SCID SHO mice as previously described^20, 39^. Treatment was performed intraperitoneally 3× per week for 2 weeks for U87MG or for 1 week for HCT116 and GBM164. The GBM164 is a patient-derived xenograft (PDX) from a 38-year-old female with a glioblastoma, harboring mutated IDH (R132H) (WHO 2007 classification). For intraperitoneal application ABT263 was dissolved in 80% Cremophor EL (SIGMA, St. Louis, MO) and 20% Ethanol (Pharmco-Aaper, Brookfield, CT) (v/v).

### Orthotopic transgenic glioblastoma and convection-enhanced delivery model

The study was approved by the Institutional Animal Care and Use Committee at the Columbia University Medical Center. PDGF-IRES-Cre virus was stereotactically injected into 6–8-week-old male or female transgenic mice (*PTEN* ^*flox/flox*^, *p53* ^*flox/flox*^, *luciferase* ^*stop-flox*^) weighing 20 to 30 g as previously described^6, 7^. A burr hole was positioned 2 mm anterior and 2 mm lateral of the bregma prior to introducing a Hamilton syringe under stereotactic guidance 2 mm into the rostral subcortical white matter. Motorized injections of the retrovirus were performed at a rate of 0.2 μl min^−1^.

Convection-enhanced delivery was performed as described before^40^. Five days after implantation of tumor cells, alzet^®^ micro-osmotic pumps (DURECT Corporation, Cupertino, CA) were implanted for a 7-day long continuous intracranial infusion of 2-HG or solvent. To verify intracranial drug administration 1% Gadolinium was added to the pumps (Omniscan, GE Healthcare Inc., Princeton, NJ) and MRIs (Bruker ICON^TM^, 1 Tesla or Bruker BioSpec^TM^, 9.4 Tesla) were performed after removal of the pump system. Survival was assessed by calculating Kaplan–Meier curves. Bioluminescent imaging was performed as described in refs ^6, 7^.

### Statistical analysis

Statistical significance was assessed by Student *t*-test using Prism version 5.04 (GraphPad, La Jolla, CA). All tests were two-sided. A *p* ≤ 0.05 was considered statistically significant. The CompuSyn software (ComboSyn, Inc., Paramus, NJ—www.combosyn.com last accessed 06/01/15) was used for the drug combination analysis including the calculation of the combination index (CI) and isobologram. A CI < 1 was considered as synergistic, a CI = 1 as additive and a CI > 1 as antagonistic. The concentration for each compound resulting in 50% inhibition (ED_50_) is normalized to 1, plotted on *x*- or *y*-axis and connected by a line, which represents the ED_50_ isobologram. Data points of drug combinations plotted below the connecting line represent a synergistic interaction, data points located on the line represent an additive interaction and data points located above the connecting line represent an antagonistic interaction.

### Data availability

The authors declare that the data within this manuscript are available in the main and/or supplementary figures or from the corresponding author upon reasonable request.

## Electronic supplementary material

Supplementary Information
