# Supplementary material for: Induction of synthetic lethality in IDH1-mutated gliomas through inhibition of Bcl-xL
Source: Nat Commun. 2017 Oct 20;8:1067. doi: 10.1038/s41467-017-00984-9 (PMC5651864; doi:10.1038/s41467-017-00984-9)
Supplement: Supplementary file 1 — Supplementary Information [file 41467_2017_984_MOESM1_ESM.pdf]

Description of Supplementary Files

File Name: Supplementary Information
Description: Supplementary Figures and Supplementary Tables

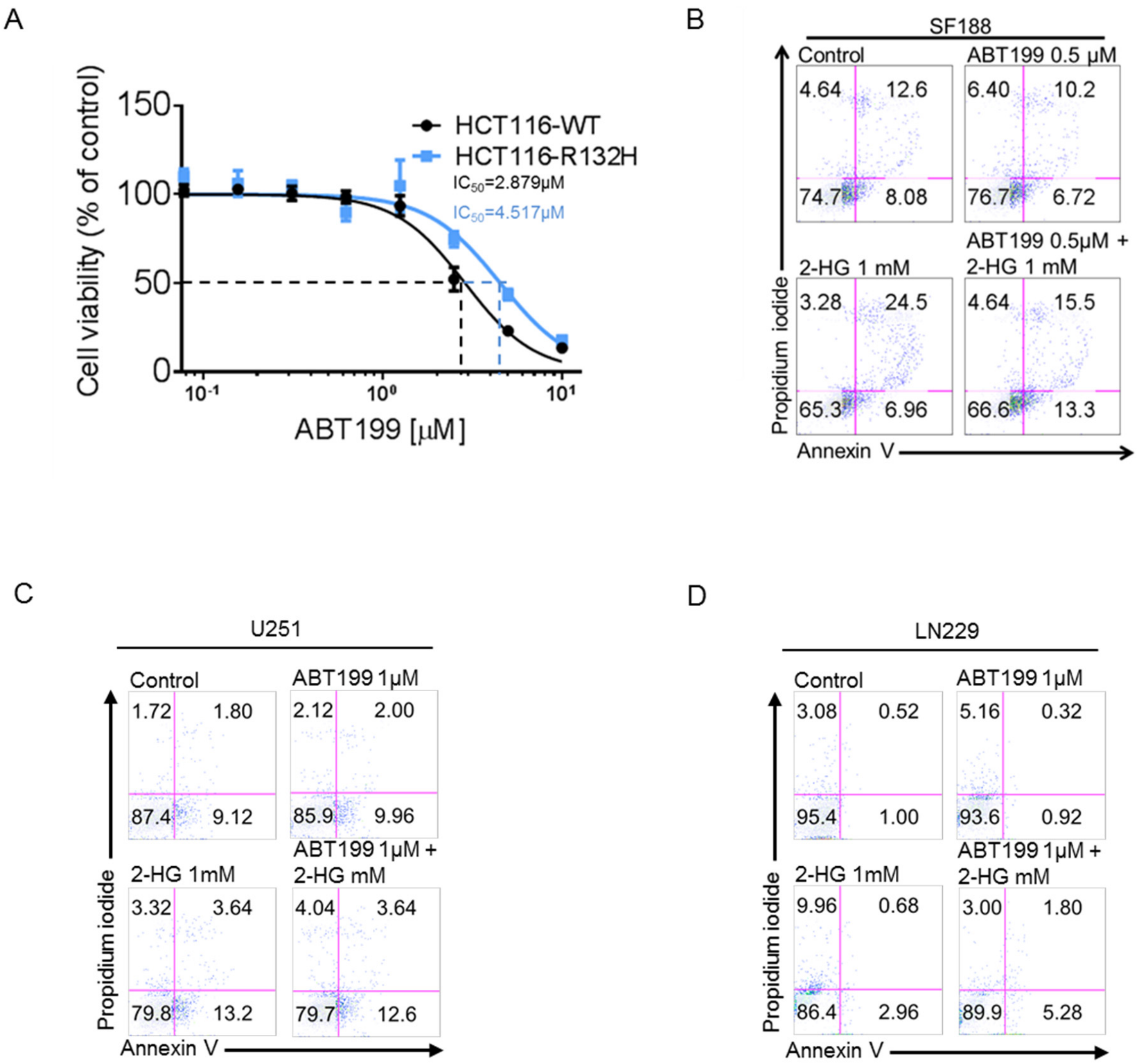

Supplementary Figure 1:

(A), HCT116 IDH1-WT and IDH1-R132H cells were treated with increasing concentrations of ABT199. After 72h of treatment cellular viability was determined via MTT assay. Non-linear regression analysis was performed to determine IC_{50}-values. Data are presented as mean and SD, n=3. **(B)**, SF188 pediatric glioblastoma cells were treated for 48h with solvent, ABT199 and 2-HG as indicated. Staining for annexin V/propidium iodide was performed prior to flowcytometric analysis. Right lower quadrant represents early apoptotic cells. Right upper quadrant represents late apoptotic or necrotic cells. Left upper quadrant represents necrotic cells. **(C-D),** U251 and LN229 cells were treated with vehicle, ABT199, 2-HG or the combination for 48h. Subsequently, cells were stained with annexin V and propidium iodide and analyzed by flow cytometry.

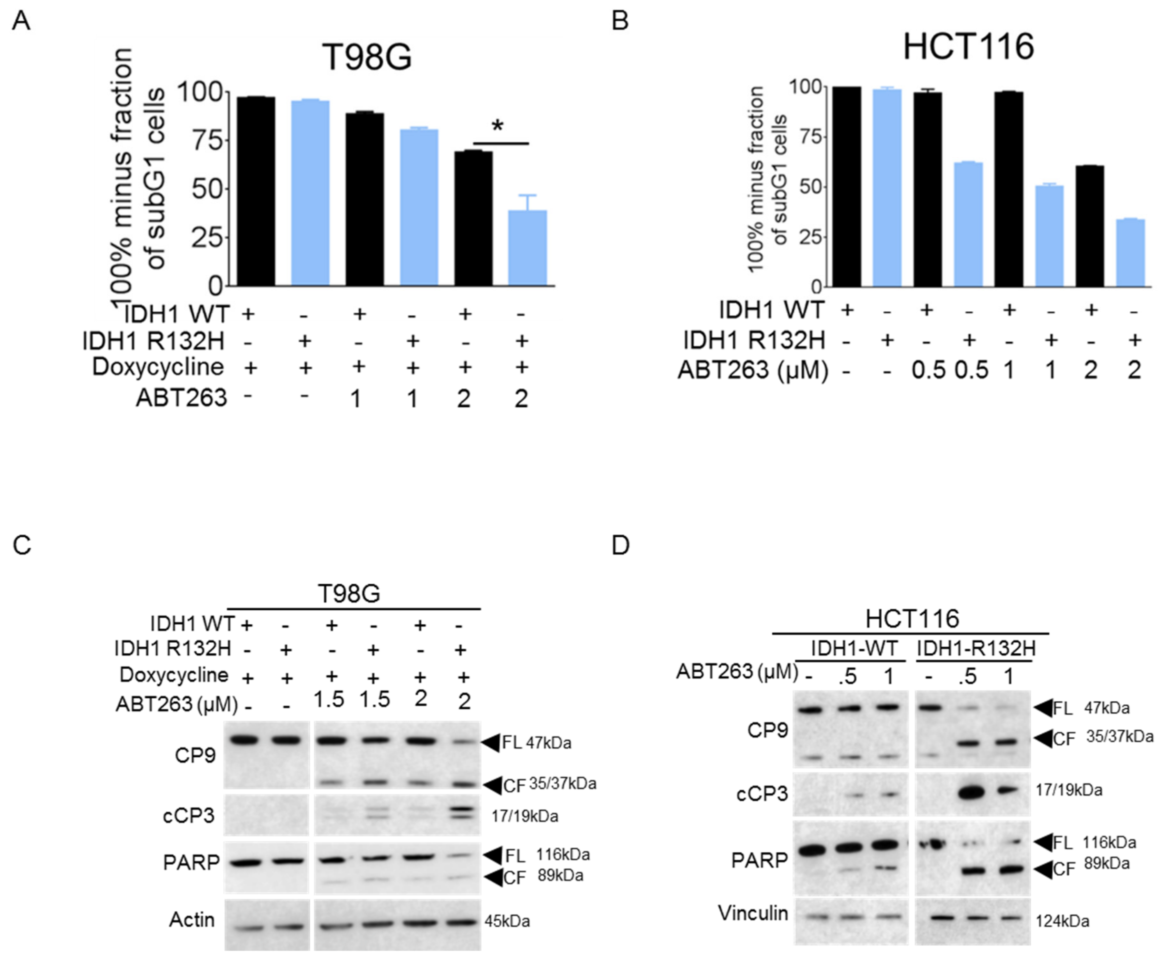

Supplementary Figure 2:

(A), T98G glioblastoma cells were retrovirally transduced with IDH1-WT or IDH1-R132H and expression was induced with doxycycline prior to treatment with solvent or ABT263 as indicated for 48h. Staining with Propidium iodide was performed and the fraction of viable cells (100%-SubG1 cells) was determined by flow cytometry. Column, mean. Error bar, SD, n=3. **(B)**, HCT116 IDH1-WT and IDH1-R132H cells were treated with solvent or ABT263 prior to staining with Propidium iodide and flow cytometric analysis. Column: mean of the fraction of viable cells. Error bar: SD, n=3. **(C)**, T98G IDH1-WT/IDH1-R132H cells were treated with ABT263 at indicated concentrations for 7h. Whole cell extracts were collected and Western blot analysis was performed for caspase 9 (CP9), cleaved caspase 3 (cCP3), PARP and Actin. FL: full length form. CF: cleaved fragment. **(D)**, HCT116 IDH1-WT/IDH1-R132H colorectal cancer cells were treated with ABT263 at indicated concentrations for 7h. Whole cell extracts were collected and Western blot analysis was performed for caspase 9 (CP9), cleaved caspase 3 (cCP3), PARP. Vinculin served as a loading control. FL: full length form. CF: cleaved fragment.

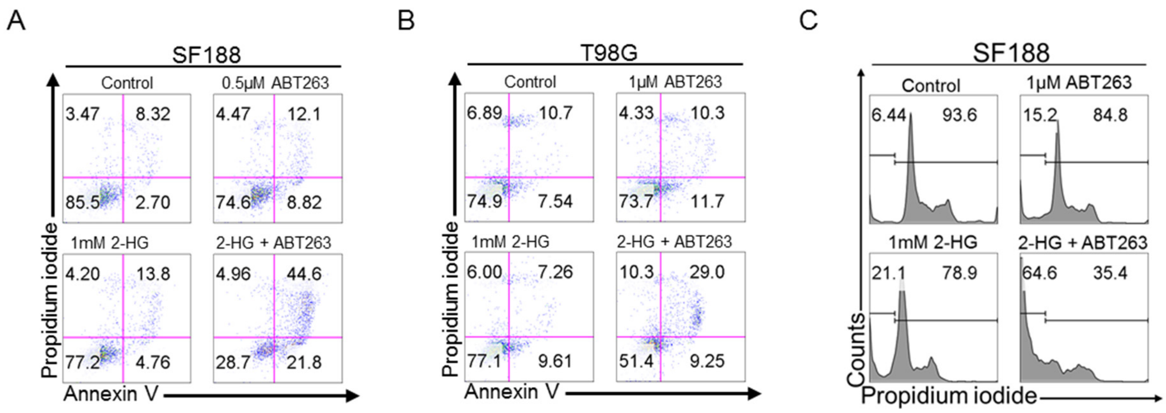

Supplementary Figure 3:

(A-B), SF188 **(A)** and T98G **(B)** glioblastoma cells were treated for 48h with solvent, ABT263 and 2-HG as indicated. Staining for annexin V/propidium iodide was performed prior to flowcytometric analysis. Right lower quadrant represents early apoptotic cells. Right upper quadrant represents late apoptotic or necrotic cells. Left upper quadrant represents necrotic cells. **(C)**, SF188 pediatric glioma cells were treated for 48h with ABT263 and 2-HG as indicated prior to staining for Propidium iodide and flowcytometric analysis. Representative flow plots are displayed.

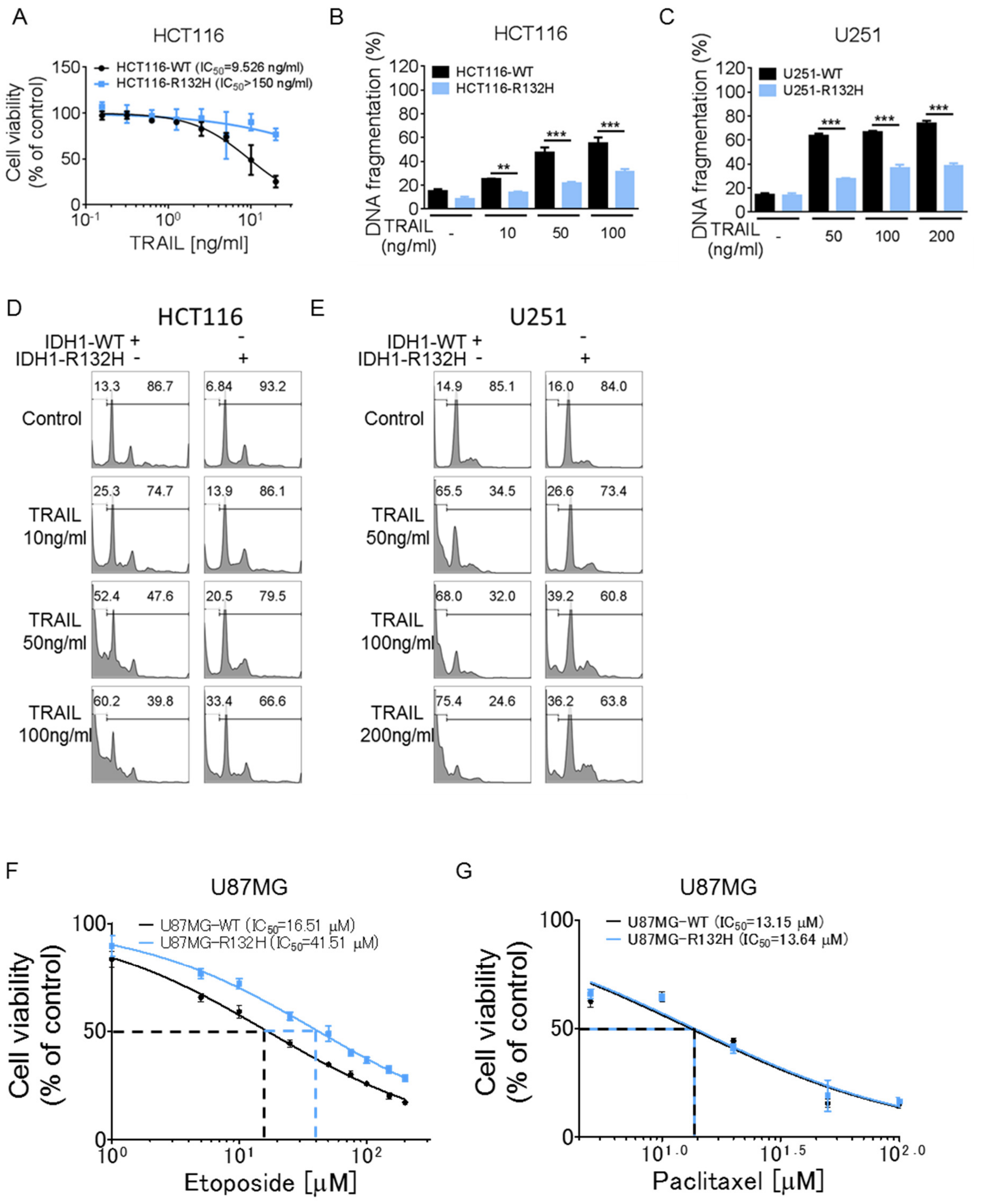

Supplementary Figure 4:

(A), HCT116 IDH1-WT and HCT116 IDH1-R132H cells were treated with increasing concentrations of TRAIL. After 72h of treatment cellular viability was determined via MTT assay. Data are presented as mean and SD, n=3. **(B-C)**, IDH1-mutated and IDH1-WT HCT116 and U251

glioma cells were treated for 72h with increasing concentrations of TRAIL. Staining for Propidium iodide was performed prior to flowcytometric analysis. Column: mean. Error bar: SD, n=3. **(D-E)**, Representative flow plots of apoptotic HCT116 and U251 cells treated as described for **(B-C)**. **(F-G)**, U87MG IDH1 wild-type or IDH1 R132H mutated cells were treated with increasing concentrations of Etoposide or Paclitaxel and analyzed for cellular viability by CellTiter-Glo® assay. Data are presented as mean and SD, n=3.

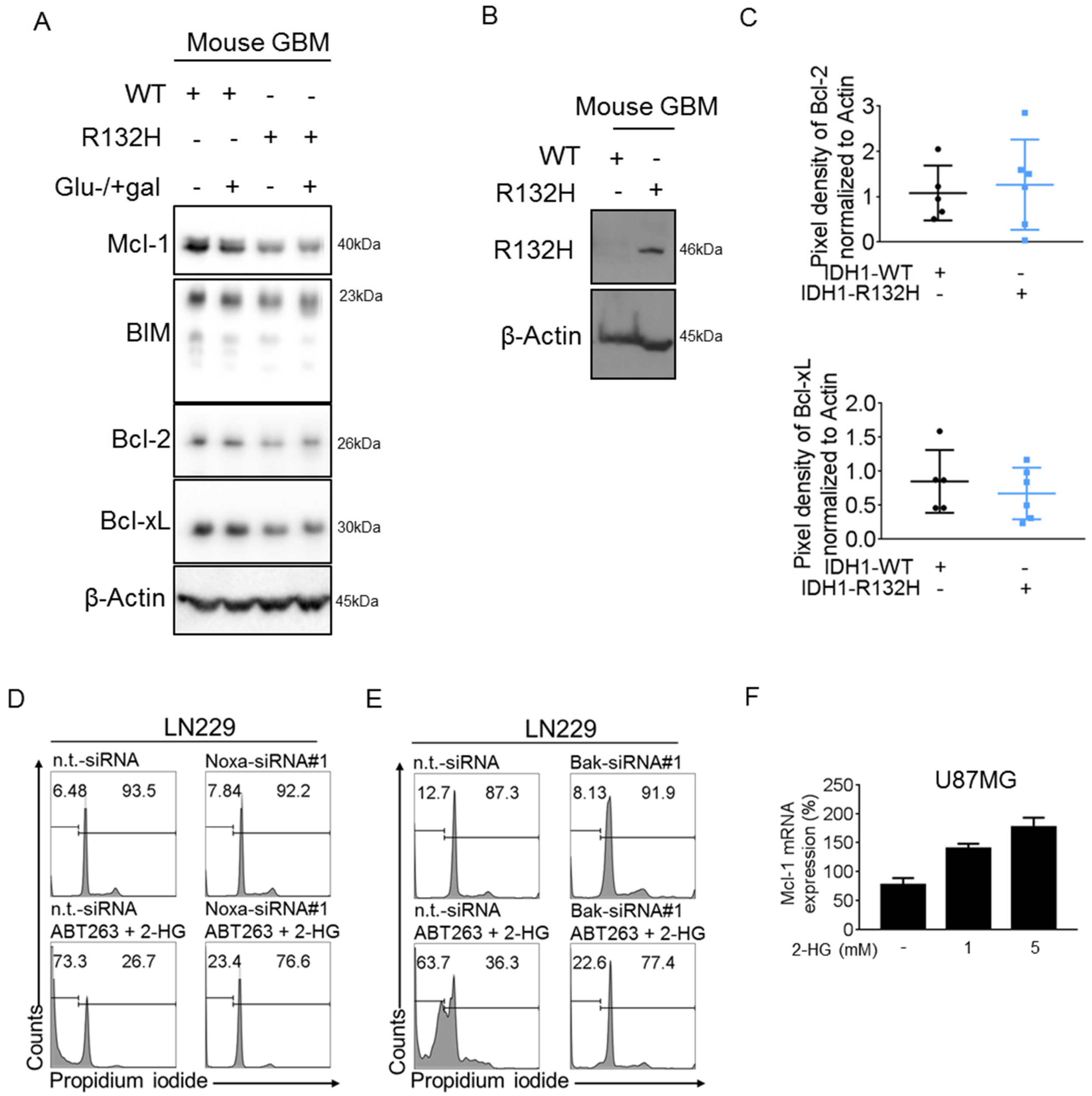

Supplementary Figure 5:

(A), Murine proneural glioblastoma cells, harboring IDH1 wild-type or IDH1 R132H, were analyzed for the expression of Mcl-1, BIM, Bcl-2, Bcl-xL and Actin by Western blot analysis either under low or high glucose conditions. **(B)**, Murine proneural glioblastoma cells, harboring IDH1 wild-type or IDH1 R132H, were analyzed for the expression of mutant IDH1. **(C)**, Scatter plot demonstrating Bcl-2 and Bcl-xL protein expression among IDH1-WT (n=5) and IDH1-R132H (n=6) expressing anaplastic astrocytoma samples (from Figure 3A). Densitometric analysis was

performed using imageJ 1.47v (http://imagej.nih.gov/ij). Pixel density was normalized to the respective Actin control. **(D)**, Representative flow plots of LN229 cells that were treated with n.t.-siRNA or Noxa-siRNA in the presence or absence of 0.5µM ABT263 and 1mM 2-HG for 48h and stained with propidium iodide to determine the fraction of apoptotic cells (subG1 fraction). (E), Representative flow plots of LN229 cells that were treated with n.t.-siRNA or Bak-siRNA in the presence or absence of 0.3µM ABT263 and 1mM 2-HG for 48h and stained with propidium iodide to determine the fraction of apoptotic cells (subG1 fraction). (F), U87MG glioblastoma cells were treated for 24h with 2-HG as indicated. Total RNA was collected and isolated prior to performing real-time rtPCR for Mcl-1 mRNA. Column: mean. Error bar: SD, n=3.

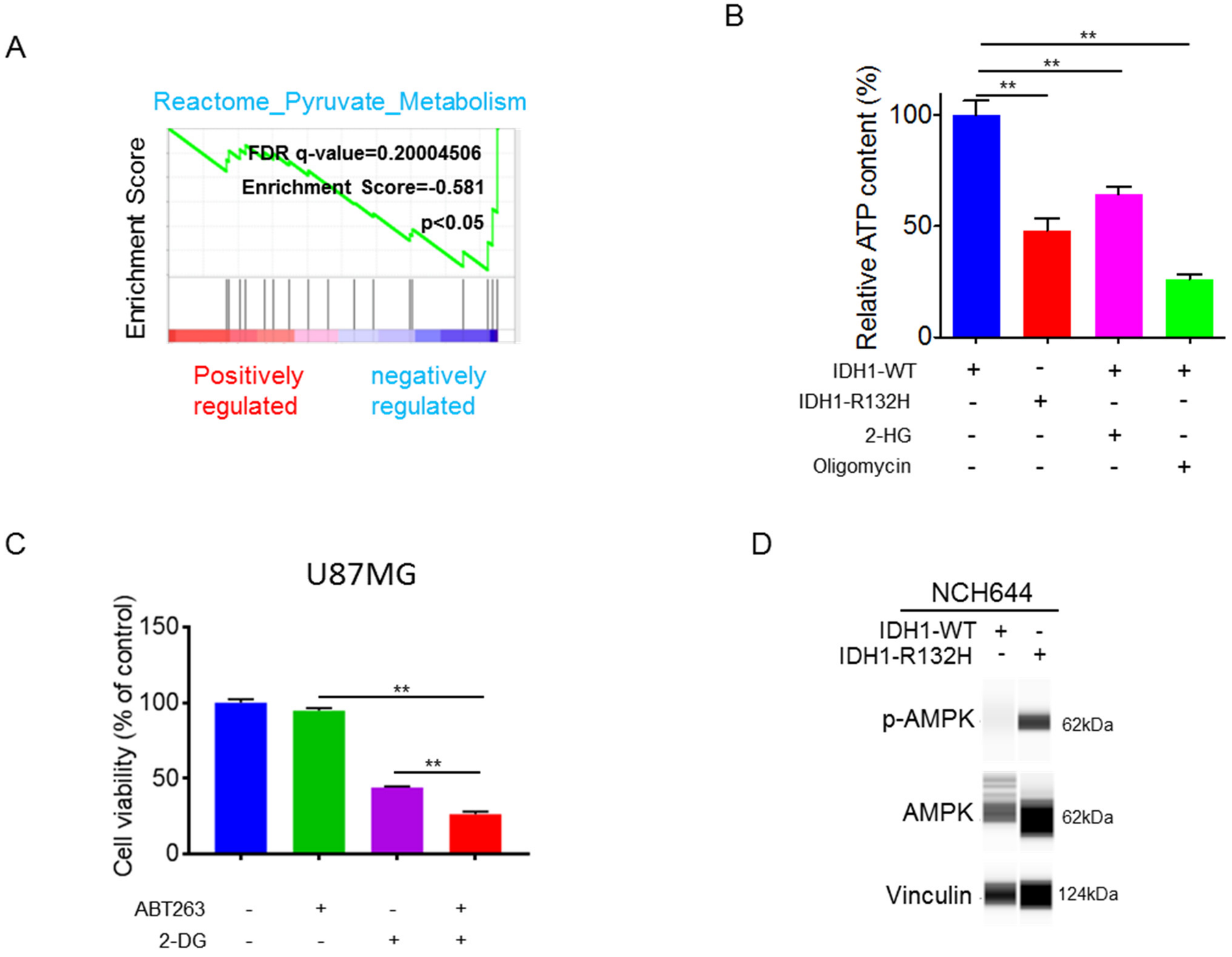

Supplementary Figure 6:

(A), Wild-type and IDH1 R132H-mutated glioblastoma cells were submitted for transcriptome analysis and subsequently analyzed by GSEA. Shown are GSEA plots with the respective statistical analysis. **(B)**, IDH1 wild-type or IDH1 R132H cells were treated as indicated with solvent, 2-DG or Oligomycin and analyzed for relative ATP content. Column: mean. Error bar: SD, n=3-4. ** indicates a p-value of less than 0.01. (Student's t-test). **(C)**, U87MG glioblastoma cells were treated with 1 µM ABT263, 5 mM 2-DG or the combination prior to analysis for cellular viability after 48h. Column: mean. Error bar: SD, n=6. ** indicates a p-value of less than 0.01 (Student's t-test). **(D)**, Whole cell extracts were collected from NCH644 IDH1 WT and IDH1 R132H glioma stem-like cells prior to capillary electrophoresis for pAMPK und AMPK. Vinculin served as a loading control.

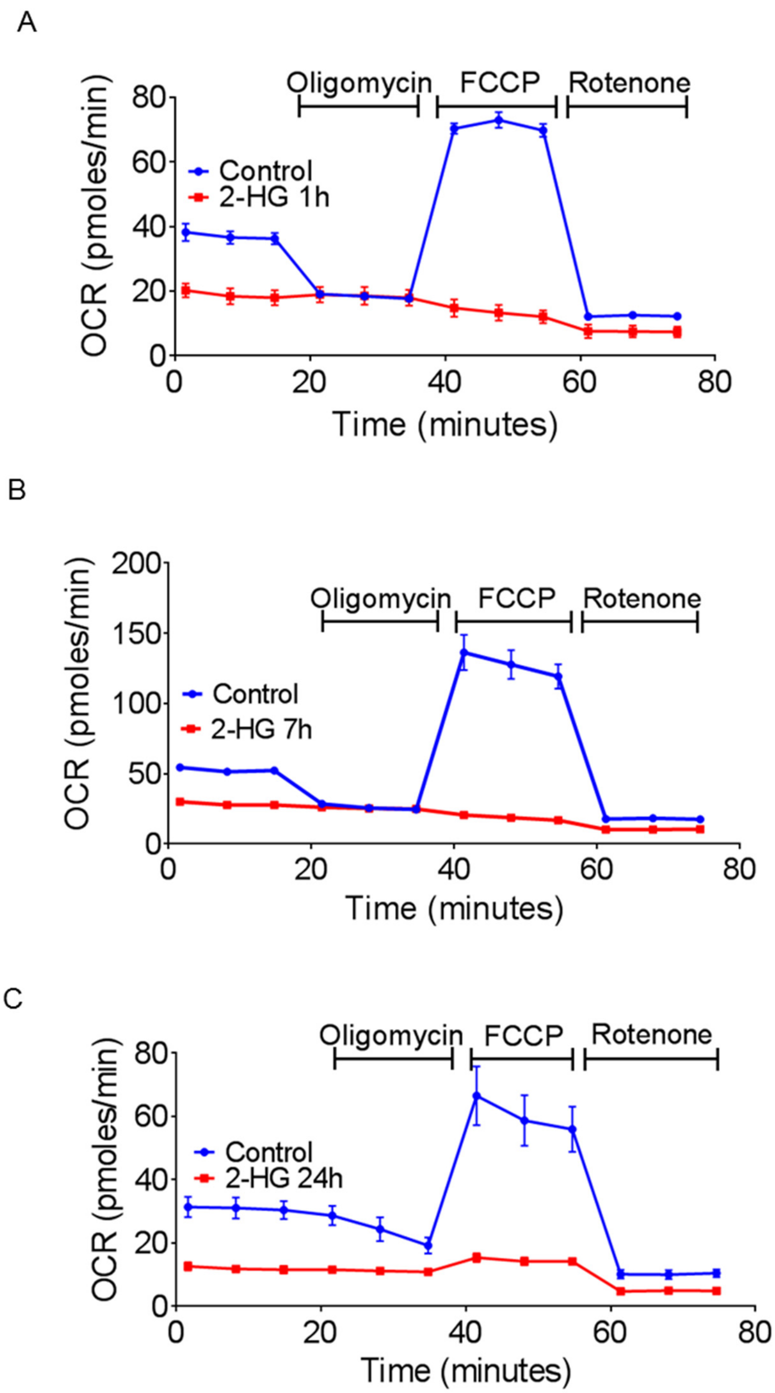

Supplementary Figure 7:

(A-C), U87MG glioblastoma cells were treated with 2-HG (1h, 7h or 24h) as indicated and analyzed for oxygen consumption rate (OCR) on a Seahorse XFp Flux analyzer in accordance with the instructions by the manufacturer (Mitochondrial Stress Kit). Data presented as mean and SD, n=3.

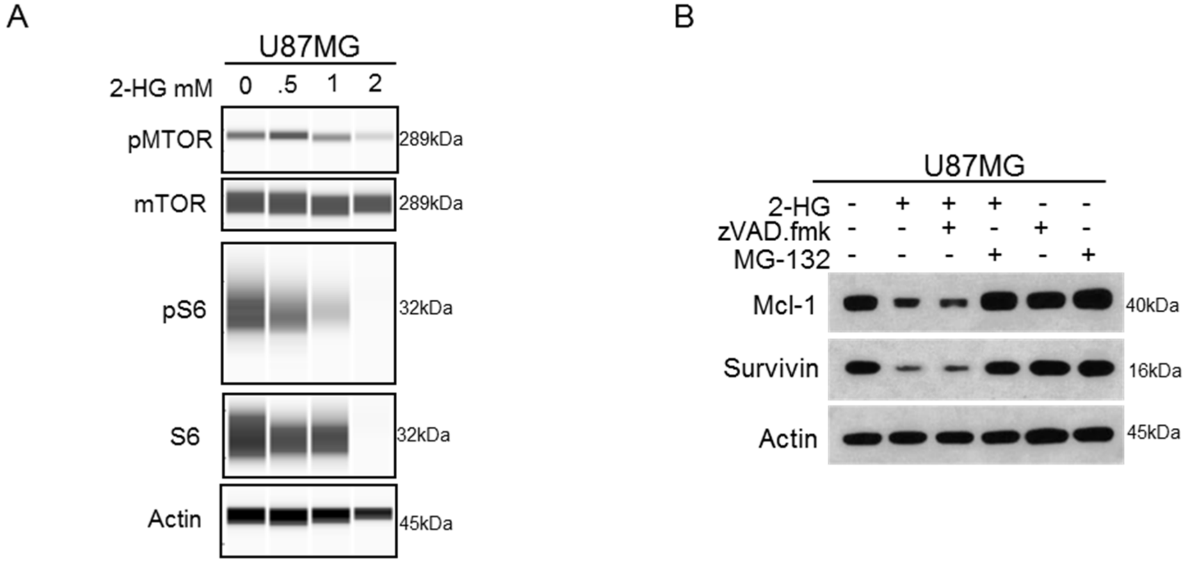

Supplementary Figure 8:

(A), U87MG glioblastoma cells were treated for 24h with 2-HG as indicated and subsequently analyzed by capillary electrophoresis for the expression of p-mTOR, mTOR, p-S6, S6 and Actin. **(B)**, U87MG cells were treated for 5h with 2-HG (1mM) in the presence or absence of the pan-caspase inhibitor zVAD.fmk (20μM) or the proteasome inhibitor MG-132 (10μM). Whole cell extracts were collected prior to performing Western blot analysis for Mcl-1 and Survivin. Actin served as control for equal loading.

Glioma stem-like cell	IC_{50} ABT263 (µM)
NCH644	4.58
NCH421K	1.83
GS9-6	1.47
BT-142	0.71
NCH612	0.21

Supplementary Table 1: IC50-values for ABT263 in IDH1-WT and IDH1-R132H glioma stem-like cells. Treatment with ABT263 results in a more pronounced response towards ABT263 among IDH1-mutated glioma stem-like cells. NCH644 (IDH1-WT), NCH412K (IDH1-WT), GS9-6 (IDH1-WT), BT-142 (IDH1-R132H) and NCH612 (IDH1-R132H) cells were treated for 72h with increasing concentrations of ABT263 prior to determining IC_{50}-values based on CellTiter-Glo assay.

U87MG			NCH644			GBM12		
**2-HG (mM)**	**ABT263 (µM)**	**CI**	**2-HG (mM)**	**ABT263 (µM)**	**CI**	**2-HG (mM)**	**ABT263 (µM)**	**CI**
2.5	2.0	0.359	2.5	10.0	0.041	1.25	0.5	0.946
2.5	0.125	0.207	2.5	0.6125	0.038	1.25	0.03	1.080
1.25	1.0	0.430	1.25	5.0	0.556	0.6	0.25	0.622
1.25	0.25	0.341	1.25	1.25	0.546	0.6	0.06	0.805
0.6	0.5	0.412	0.6125	2.5	1.497	0.3	0.125	0.720
0.3	1.0	0.744	0.30625	1.25	1.218	0.15	0.25	1.318
0.3	0.25	0.897	0.15	10.0	0.853	0.15	0.06	0.763

Supplementary Table 2: Combination index-values for a combined treatment with 2-HG and ABT263. ABT263 treatment yields a synergistic antiproliferative effect in the presence of 2-HG. The CompuSyn software (ComboSyn, Inc., Paramus, NJ) was used for the drug-drug interaction analysis including the calculation of the combination index (CI). A CI<1 was considered as synergistic, a CI=1 as additive and a CI>1 as antagonistic.

Gene	Forward sequence	Reverse sequence
Mcl-1	CCA AGA AAG CTG CAT CGA ACC AT	CAG CAC ATT CCT GAT GCC ACC T
USP9X	GTG TCA GTT CGT CTT GCT CAG C	GCT GTA ACG ACC CAC ATC CTG A
GAPDH	GTC TCC TCT GAC TTC AAC AGC G	ACC ACC CTG TTG CTG TAG CCA A
18S	AGT CCC TGC CCT TTG TAC ACA	GAT CCG AGG GCC TCA CTA AAC

Supplementary Table 3: Primer sequences for real-time PCR analysis of mRNA levels.

Figure 2A

Bcl-xL

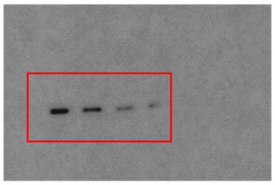

Figure 2A

Actin

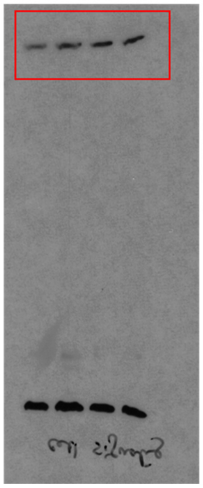

Figure 2B

Bcl-xL

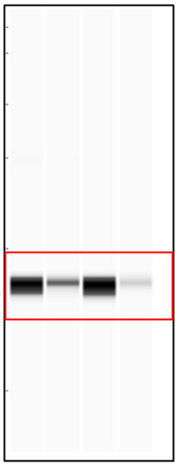

Figure 2B

Vinculin

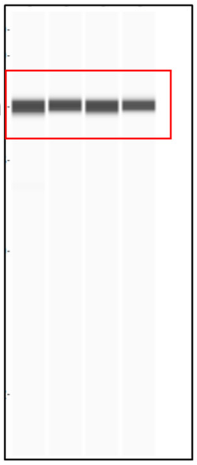

Figure 2C

Actin

Bcl-xL

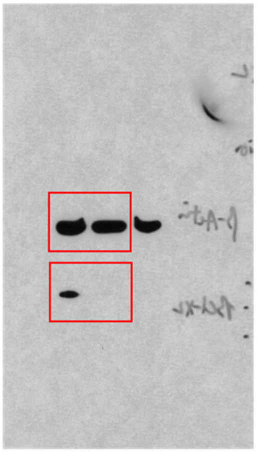

Figure 2D

Bcl-xL

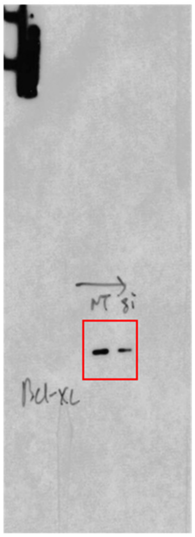

Figure 2D

Actin

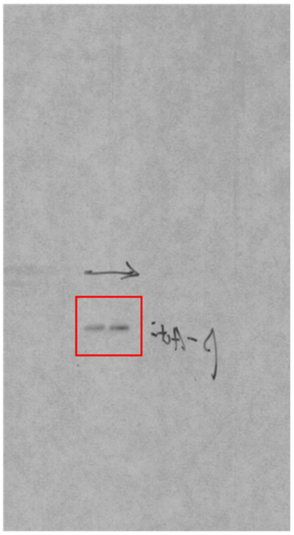

Figure 2G left panel

CP9

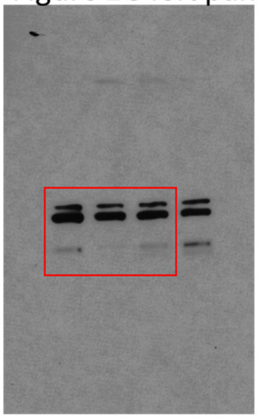

Figure 2G left panel

PARP

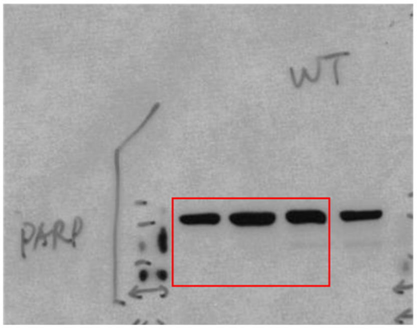

Figure 2G left panel

cCP3

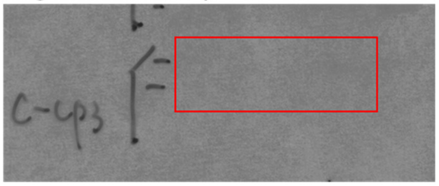

Figure 2G left panel

Actin

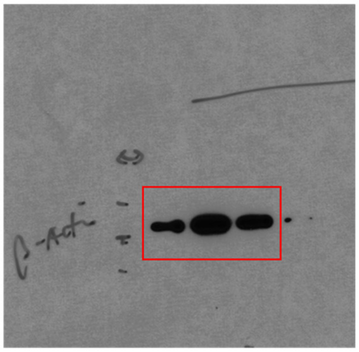

Figure 2G right panel

CP9

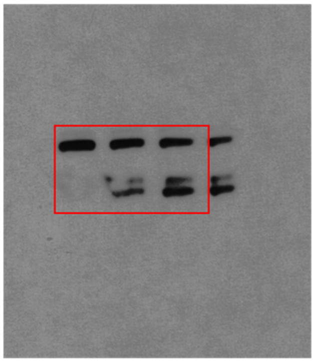

Figure 2G right panel

PARP

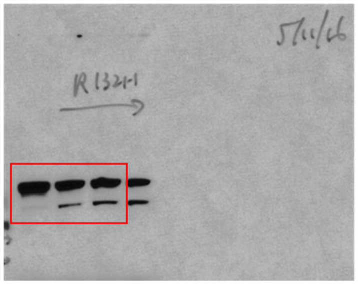

Figure 2G right panel

cCP3

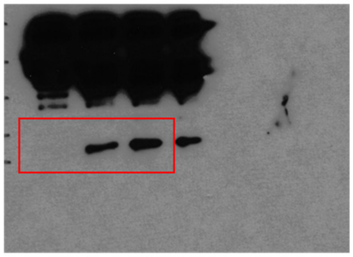

Figure 2G right panel

Actin

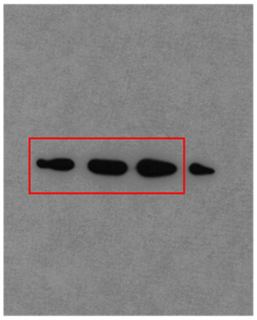

Figure 2J left panel

cPARP

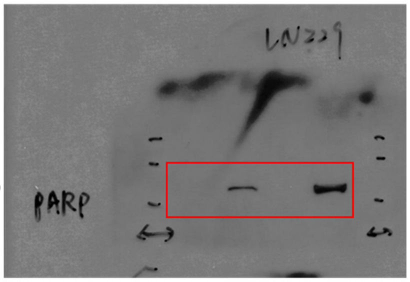

Figure 2J left panel

CP9

cCP3

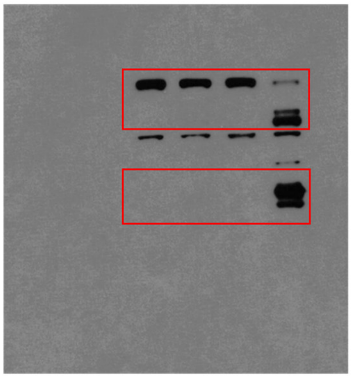

Figure 2J left panel

Actin

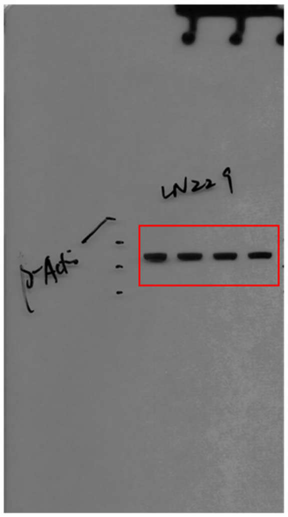

Figure 2J right panel

cPARP

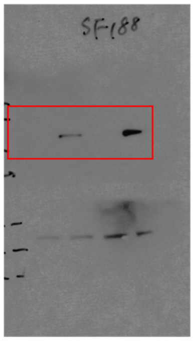

Figure 2J right panel

CP9

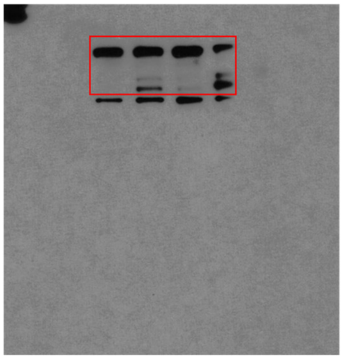

Figure 2J right panel

cCP3

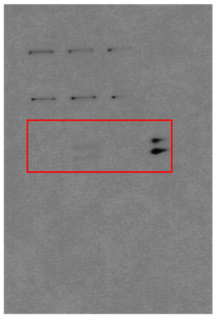

Figure 2J right panel

Actin

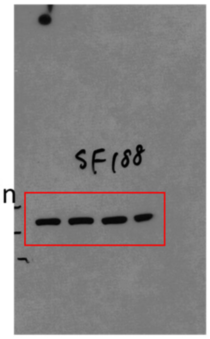

Figure 3A

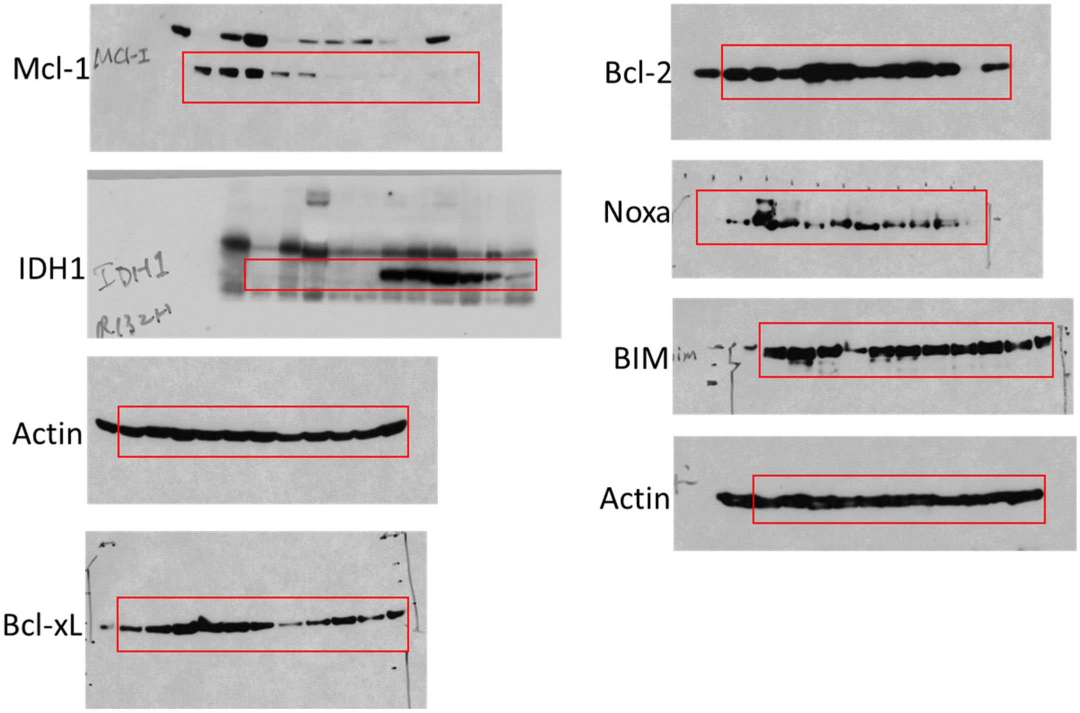

Figure 3C

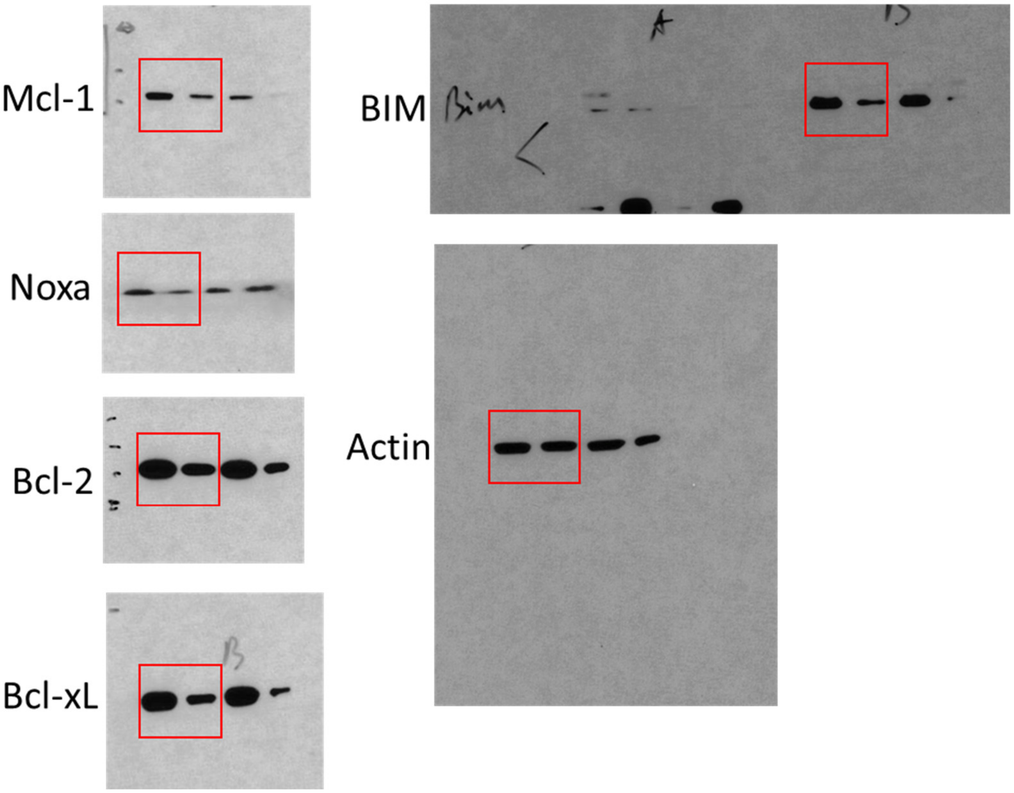

Figure 3D

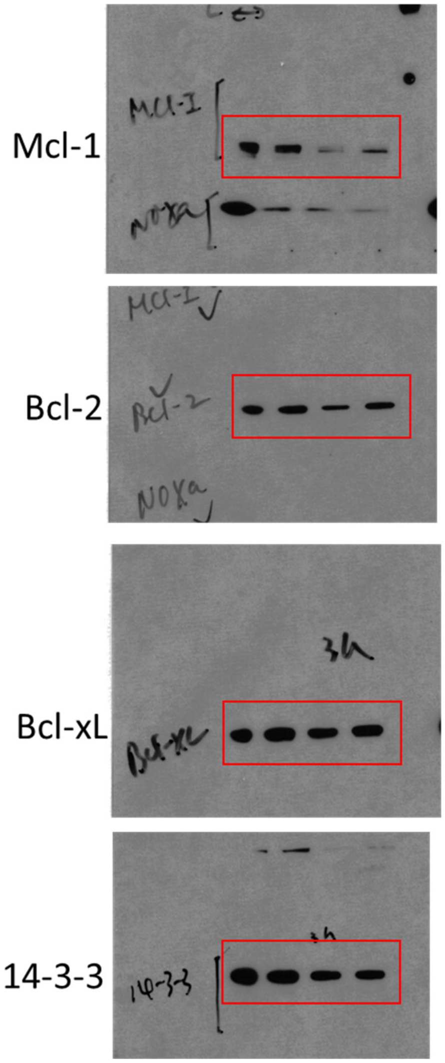

Figure 3E

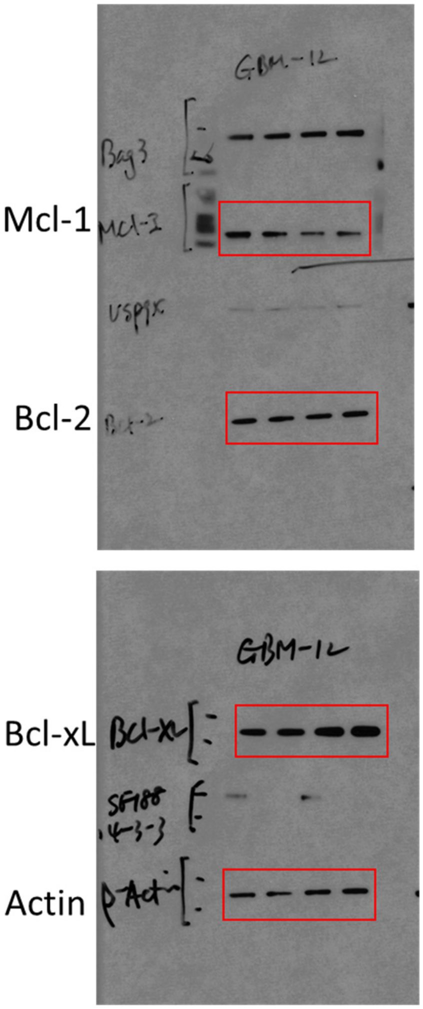

Figure 3F

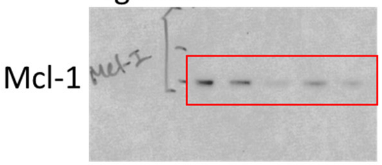

Figure 3F

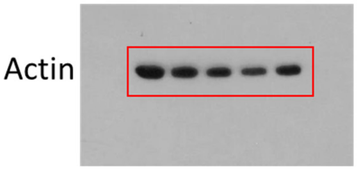

Figure 3G

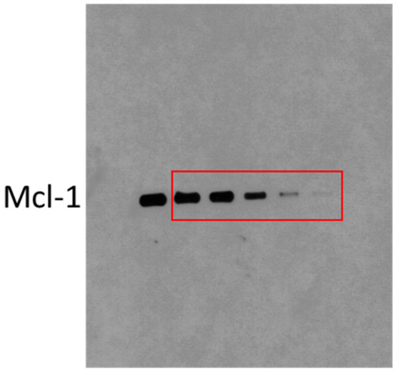

Figure 3G

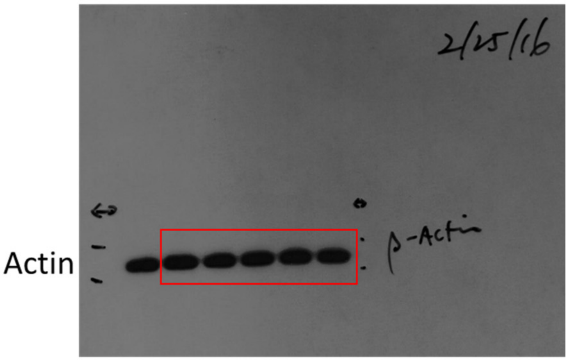

Figure 3H

Noxa

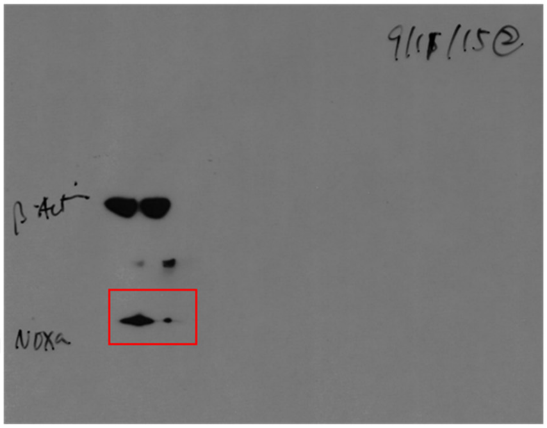

Figure 3I

BAK

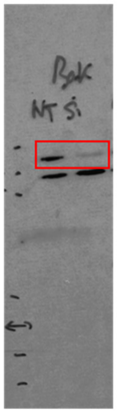

Figure 3H

Actin

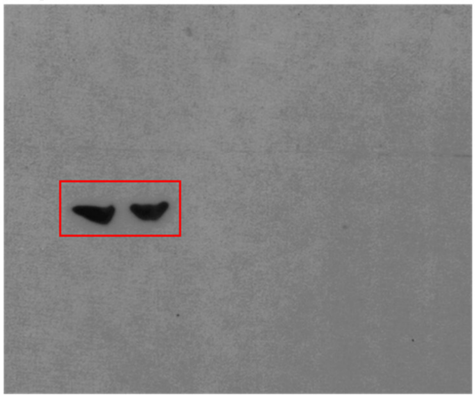

Figure 3I

Actin

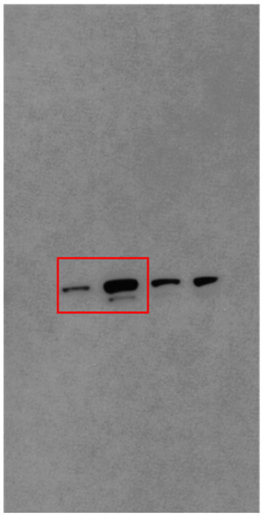

Figure 4I left panel

Mcl-1

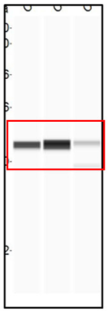

Figure 4I right panel

Mcl-1

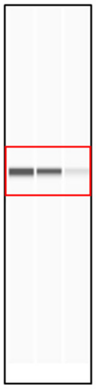

Figure 4I left panel

Vinculin

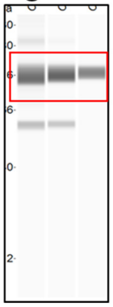

Figure 4I right panel

Vinculin

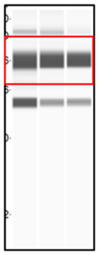

Figure 5A

Puromycin

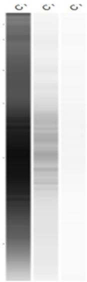

Figure 5B

Puromycin

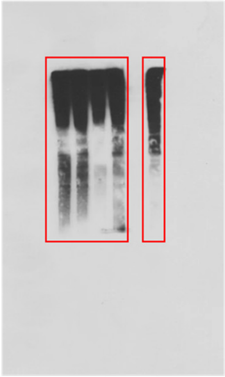

Figure 5C

pAMPK

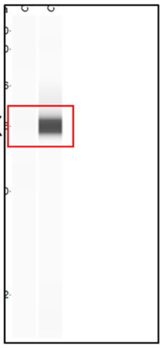

Vinculin

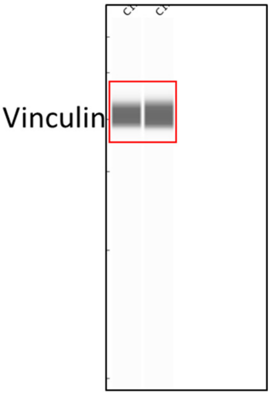

Figure 5A

Vinculin

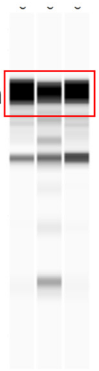

Figure 5B

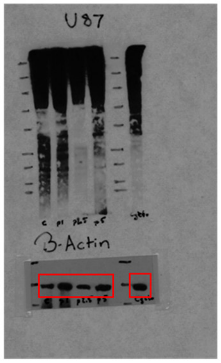

Actin

Figure 5C

AMPK

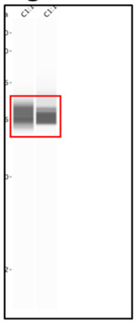

Figure 5D

pMTOR

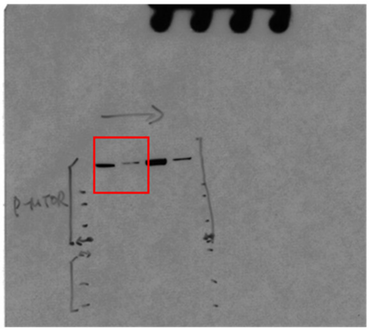

Figure 5D

p4EBP1

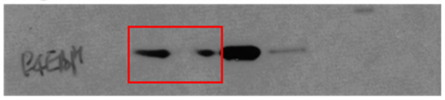

Figure 5D

pS6

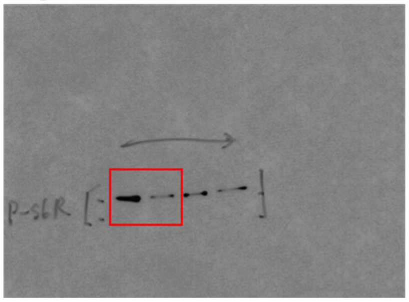

Figure 5D

Actin

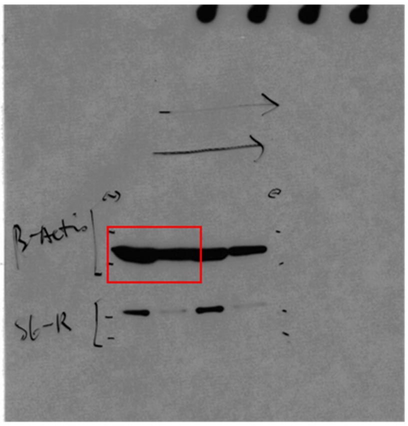

Figure 5E left panel

pAMPK

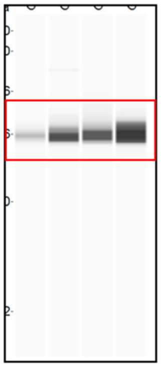

Figure 5E right panel

pAMPK

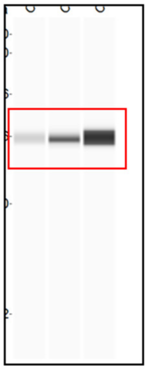

Figure 5E left panel

AMPK

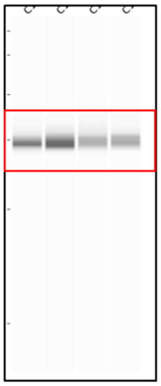

Figure 5E right panel

AMPK

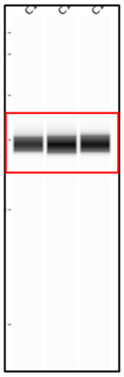

Figure 5F

p-mTOR

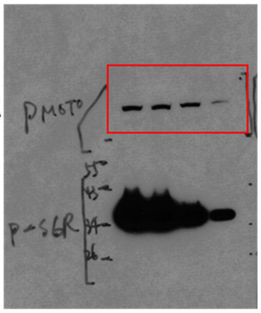

S6

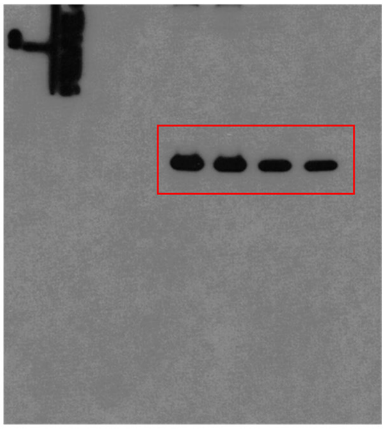

Figure 5F

mTOR

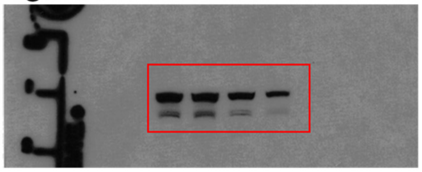

Figure 5F

pS6

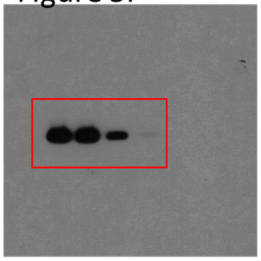

Figure 5F

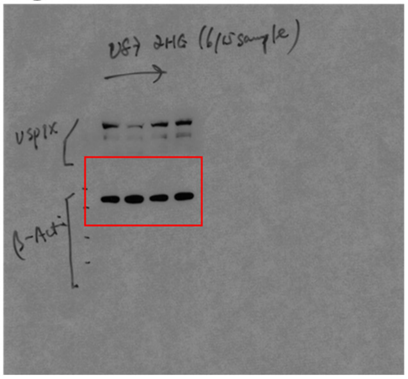

Figure 5G

p-mTOR

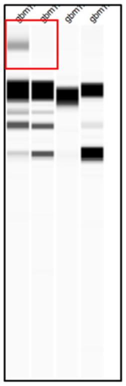

Figure 5G

p-S6

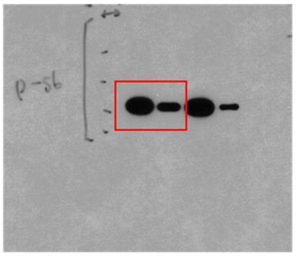

Figure 5G

mTOR

Vinculin

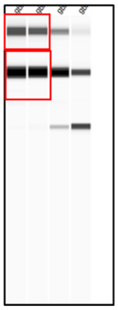

Figure 5G

S6

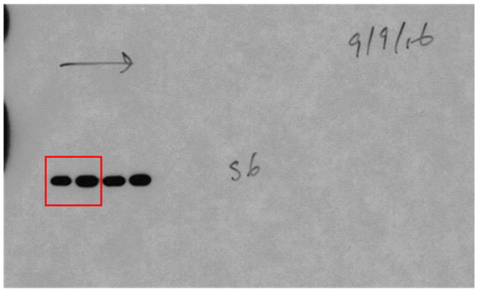

Supplementary Figure 2C

CP9

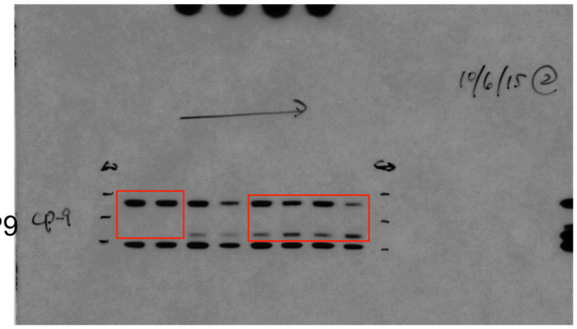

Supplementary Figure 2C

PARP

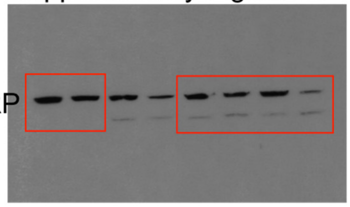

Supplementary Figure 2C

cCP3

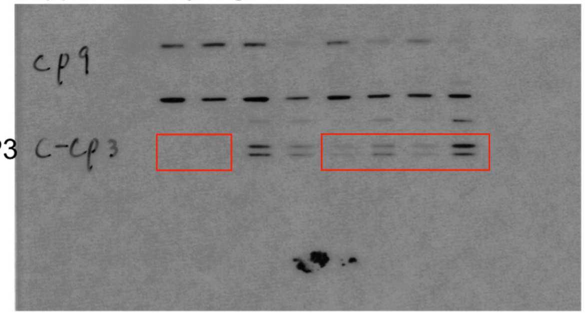

Supplementary Figure 2C

Actin

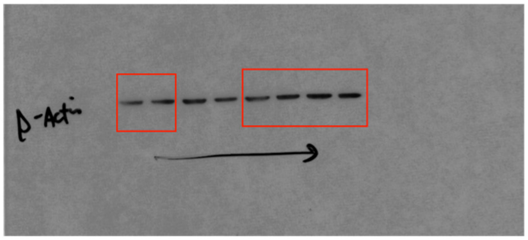

Supplementary Figure 2D left panel

CP9

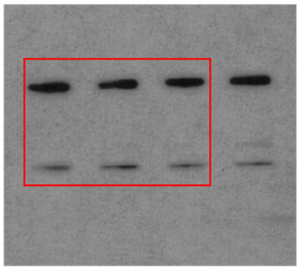

Supplementary Figure 2D left panel

cCP3

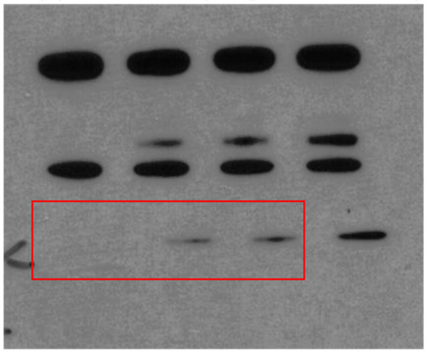

Supplementary Figure 2D left panel

PARP

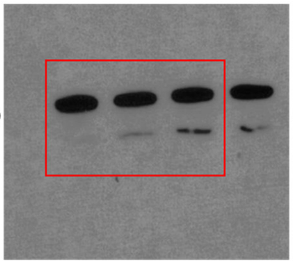

Supplementary Figure 2D left panel

Vinculin

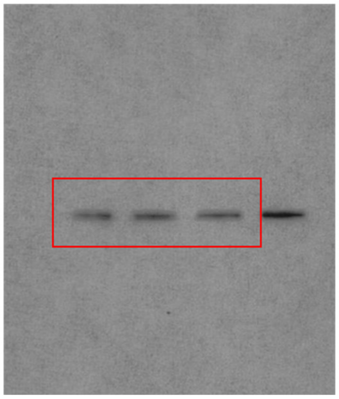

Supplementary Figure 2D right panel

PARP

CP9

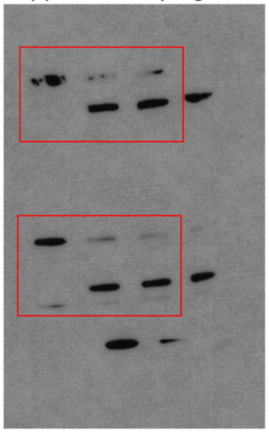

Supplementary Figure 2D right panel

cCP3

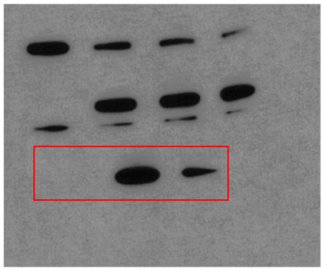

Supplementary Figure 2D right panel

Vinculin

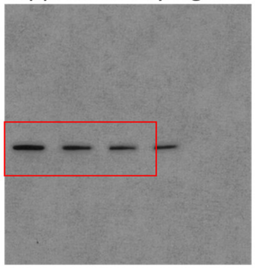

Supplementary Figure 5A

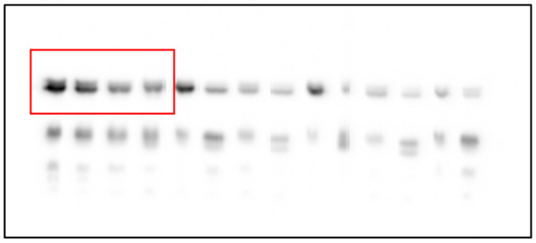

Supplementary Figure 5A

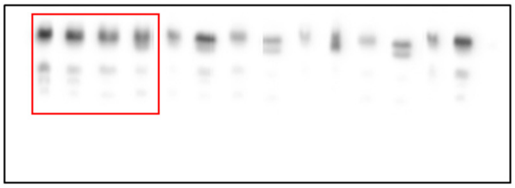

Supplementary Figure 5A

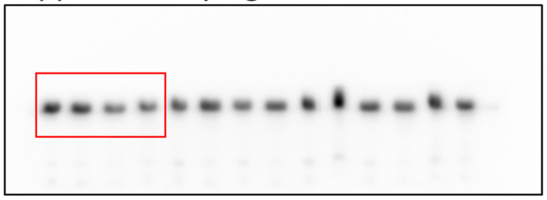

Supplementary Figure 5A

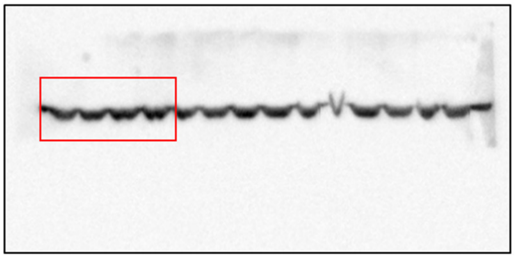

Supplementary Figure 5A

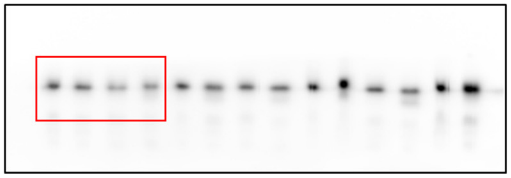

Supplementary Figure 5B

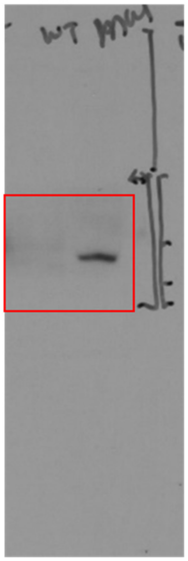

Supplementary Figure 5B

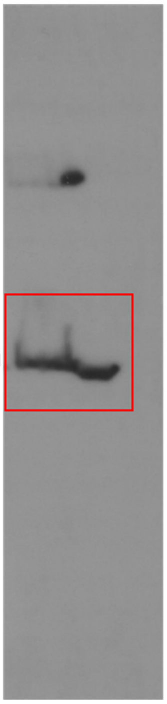

Supplementary Figure 6D

p-AMPK

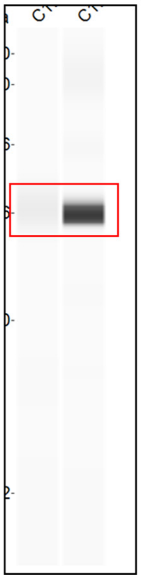

Supplementary Figure 6D

AMPK

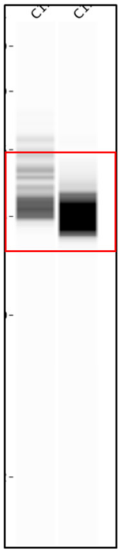

Supplementary Figure 6D

Vinculin

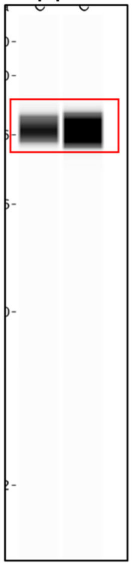

Supplementary Figure 8A

pMTOR

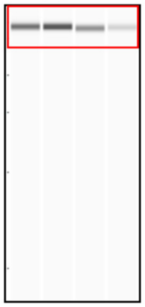

Supplementary Figure 8A

pS6

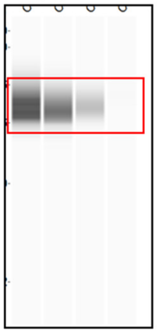

Supplementary Figure 8A

Actin

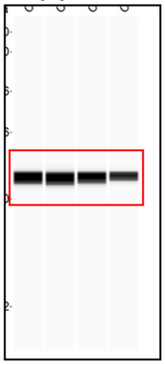

Supplementary Figure 8A

mTOR

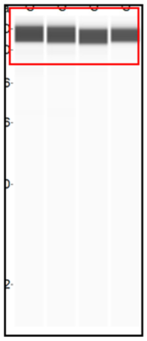

Supplementary Figure 8A

S6

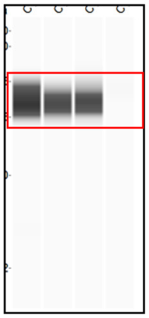

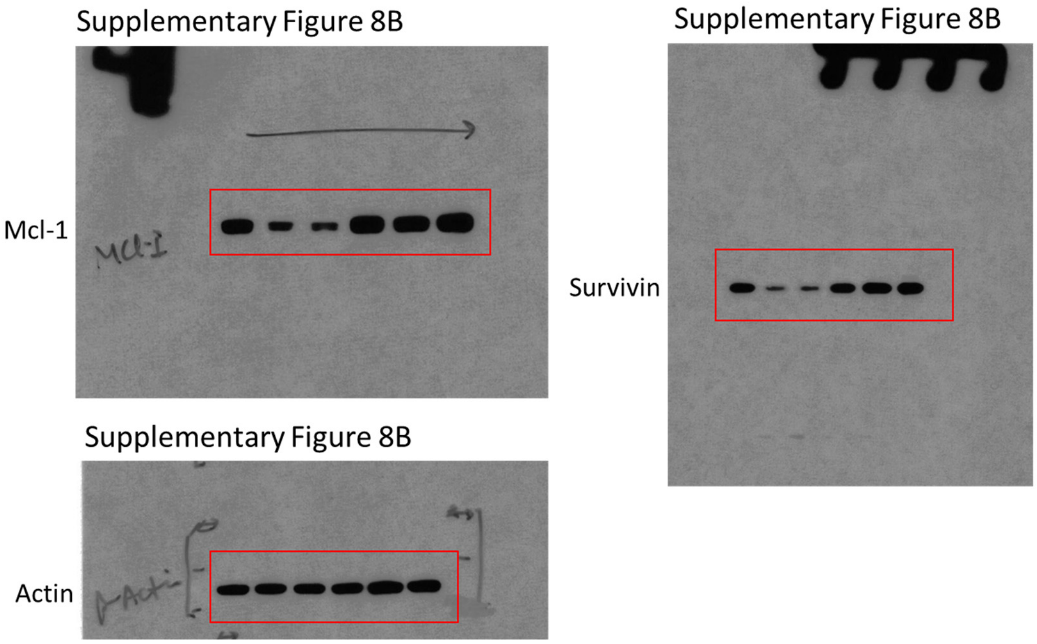

Supplementary Figure 9:

Western blot and capillary electrophoresis data. Uncropped western blots and capillary electrophoresis data from both the main and supplementary figures.
